# Distinct neural mechanisms underlying cognitive difficulties in preterm children born at different stages of prematurity

**DOI:** 10.1016/j.nicl.2025.103876

**Published:** 2025-09-03

**Authors:** Samson Nivins, Nelly Padilla, Hedvig Kvanta, Gustaf Mårtensson, Ulrika Ådén

**Affiliations:** a Department of Women's and Children's Health, Karolinska Institutet, Stockholm, Sweden; b Department of Biomedical and Clinical Sciences, Linköping University, Linköping, Sweden

**Keywords:** Preterm birth, Brain development, Lower cognitive performance, ASD, ADHD, Altered brain structures, SCN

## Abstract

**Objectives:**

To examine associations between low cognitive-performance and regional-and network-level brain changes at ages 9–10 in very-preterm, moderately-preterm, and full-term children, and explore whether these alterations predict ASD/ADHD symptoms at age 12.

**Methods:**

This longitudinal population-based study included 9–10-year-old U.S. children from ABCD Study. Children underwent brain imaging and cognitive assessment using NIH Toolbox. Cortical thickness and subcortical volumes of preterm-children with low cognitive-performance (NIH composite score < -1SD and > -2SD) were compared with preterm and full-term peers with typical performance (≥-1SD). Structural covariance networks were also examined.

**Results:**

Among 7281 children (mean age 9.9 ± 0.6 years; 52.2 % boys), 71 were very-preterm, 151 moderately-preterm, and 7056 full-term. Low cognitive-performance was most prevalent in very-preterm children (29.6 %), followed by moderately-preterm (24.0 %) and full-term children (16.2 %).

Very-preterm children with low cognitive-performance had thinner inferior temporal cortex (β = -0.58; p = 0.03), thinner fusiform gyrus (β = -0.62; p = 0.02), and larger amygdala volumes (β = 0.41; p = 0.05) compared to very-preterm children with typical performance. Moderately-preterm children with low cognitive-performance had smaller hippocampal volumes (β = -0.32; p = 0.01). Similar patterns were observed when comparing preterm children with low cognitive-performance to full-term peers with typical performance. Structural covariance network analysis revealed stronger covariance between the precuneus-postcentral gyrus pair among moderately-preterm children with low cognitive-performance. Individualized Differential Structural Covariance Network values extracted from this pair were positively associated with ASD/ADHD symptoms, though not statistically significance.

**Conclusion:**

Low cognitive performance in preterm children is associated with distinct regional and network-level brain differences, differing by prematurity. Stronger hub covariance may reflect compensatory mechanisms, highlighting the need for prematurity-tailored interventions.

## Highlights

• Preterm birth disrupts brain development and increases the risk of cognitive difficulties in childhood and beyond.
• Cognitive outcomes after preterm birth vary, with some children facing lasting challenges and others developing typically.
• Neural mechanisms underlying these divergent cognitive trajectories in preterm children remain poorly understood.
• Preterm children with lower cognition show distinct brain and network changes vs. preterm peers with typical cognition.
• Differences vary by prematurity level, suggesting compensatory mechanisms and need for tailored interventions.

## Graphical abstract

**Figure:**
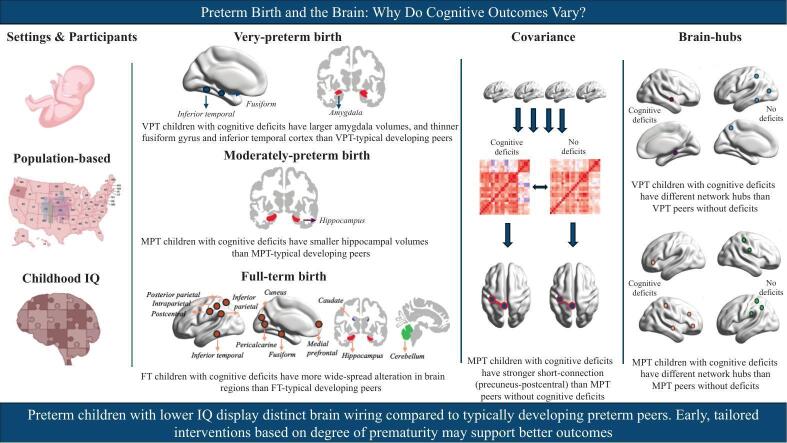

## Nomenclature

**Glossary**
SCN: Structural Covariance Network
ASD: Autism spectrum disorder
ADHD: Attention-deficit/hyperactivity disorder
ROI: Region-of-interest
IDSCN: Individualized Differential Structural Covariance Network
ABCD: Adolescent Brain and Cognitive Development
SSRS: Short-Social Responsive Scale
CBCL: Child Behaviour Checklist
SES: Socioeconomic status
VPT-Low cognitive: Very-preterm children with low cognitive performance
VPT-Typical: Very-preterm children with typical cognitive performance
MPT- Low cognitive: Moderately-preterm children with low cognitive performance
MPT-Typical: Moderately-preterm children with typical cognitive performance
FT-Typical: Full-term children with typical cognitive performance

## 1 Introduction

Preterm birth (<37 weeks) is recognized as a risk factor for low cognitive performance during school-age and beyond, with those born before 34 weeks at even higher risk (Husby et al., 2023, Nivins et al., 2025), which are, in turn, associated with poorer educational performance in adulthood.

Brain development begins as early as third week of gestation and accelerates during third trimester (Clouchoux et al., 2012), involving multiple biological processes such as myelination, neuronal organization, spinogenesis, and synaptogenesis, all of which are essential for cognitive function. Preterm birth disrupts these developmental events, prematurely exposing immature brain to extrauterine conditions, potentially altering cognitive development across postnatal ages.

Numerous studies have reported altered cortical and subcortical development in very (<32 weeks) or moderately (32–34 weeks) preterm children during mid-childhood (Lax et al., 2013, Soria-Pastor et al., 2009, Zhang et al., 2015), particularly in frontal and parietal cortices and in thalamus, hippocampus, amygdala, and cerebellum (Chau et al., 2019). These changes have also been associated with low cognitive performance (Soria-Pastor et al., 2009). However, most of change rely on correlation or case-control designs, which do not fully capture the variability in cognitive outcomes among preterm children (Brydges et al., 2018, Linsell et al., 2018). Although, on average, preterm children tend to show delays in cognitive development, there is considerable heterogeneity, with some experiencing difficulties while others do not (Brydges et al., 2018, Linsell et al., 2018). Direct comparisons between children with low versus typical cognitive performance in preterm and full-term groups remain limited yet are essential for identifying similar or distinct neural mechanisms for recognizing at-risk children and developing targeted interventions.

Individual brain regions are highly interdependent, mature in a coordinated manner within large-scale networks (Mechelli et al., 2005). Studying them in isolation, without considering network-level changes, limits our understanding of how preterm birth affects brain development and cognition. From a developmental perspective, brain network organization progresses from strengthening local connections to establishing long-range connections between distant regions (Cao et al., 2017), mirroring trajectory of cognitive development (Johnson and Munakata, 2005).

Structural Covariance Network (SCN) analysis, a multivariate method that assesses how morphological properties of different brain regions co-vary at group level (Alexander-Bloch et al., 2013). Conceptually similar to functional connectivity, regions that co-vary structurally are often part of same functional network, influenced by shared developmental mechanisms, trophic interactions, and experience-driven plasticity. SCN analyses have been widely used in autism spectrum disorder (ASD) and attention-deficit/hyperactivity disorder (ADHD) (Bethlehem et al., 2017), and have also been applied to examine preterm brain alterations in adolescence (Nosarti et al., 2011).

In this study, we used conventional region-of-interest (ROI) and SCN analyses to investigate regional and network level brain morphometry in children with low versus typical cognitive performance, separately for very- and moderately-preterm and full-term groups at 9–10 years. We focused on primary-sensorimotor and higher-order networks (Sa de Almeida et al., 2021), which are critical for cognitive development and disproportionately affected by preterm birth. Primary-sensory networks involved in basic sensory and motor processing, develop earlier and provide a scaffold for maturation of higher-order networks responsible for attention, memory, and executive function. Given that low cognitive performance in preterm children often are associated with symptoms characteristic of ASD and ADHD (James et al., 2018), we applied Individualized Differential Structural Covariance Network (IDSCN) analysis (Liu et al., 2021), an approach that identifies structural covariance differences at an individual level. We assessed whether these aberrations in preterm children with low cognitive performance could predict ASD and ADHD symptoms at 12 years. We hypothesized that preterm children with low cognitive performance would exhibit distinct neural mechanism, with stronger covariance in primary-sensory and weaker covariance in higher-order networks, potentially predicting later ASD and ADHD symptoms.

## 2 Methods

All participants were part of the Adolescent Brain and Cognitive Development (ABCD; Curated Annual Release 5.0) study, a prospective and longitudinal study of 11,875 children aged 9–10 years recruited between September 2016 and August 2018 from 21 sites across the U.S. (Garavan et al., 2018). Eligibility criteria excluded children with severe intellectual, sensory, medical, neurological conditions, as well as those born extremely preterm (<28 weeks) or with low birth weight (<1200 g), to adhere to the study protocol. Parents/guardians provided written consent, and children gave assent. All data were deidentified. The complete study protocol was approved by Swedish Ethical Review Authority (Etikprövningsmyndigheten) and conducted in accordance with Swedish ethical guidelines (Dnr 2025–03691-01). The present study followed the Strengthening the Reporting of Observational Studies in Epidemiology **(STROBE)** reporting guideline. Data were analysed from Nov 2024 to January 2025.

**Fig. 1:**
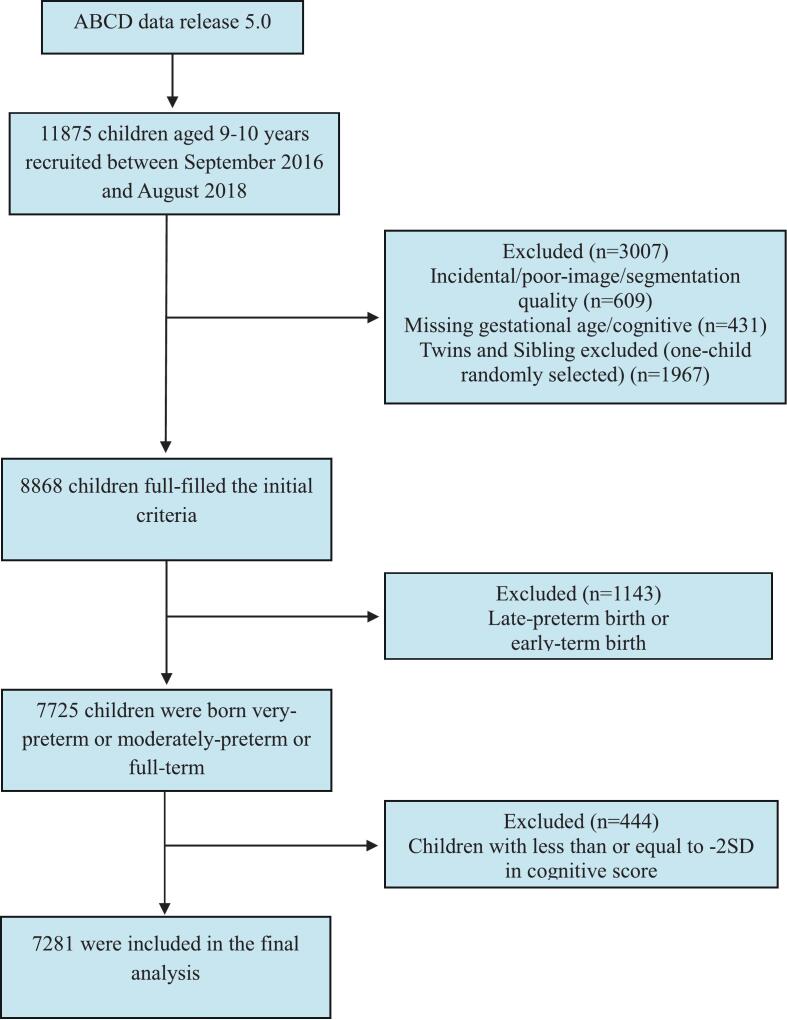
STROBE flow chart of the study participants.

Participants completed in-person assessments, including clinical interviews, surveys, neurocognitive tests, and brain imaging (Barch et al., 2018). In this study, children with missing data on gestational age, cognitive measures, or brain imaging were excluded from the analysis **(**Fig. 1**)**. Since the ABCD study included twins and siblings, one child per family was randomly selected to prevent bias.

### 2.1 Structural brain MRI

Three-dimensional T1-weighted images, acquired using 3-Tesla magnetic resonance imaging (MRI) scanners were corrected for distortions, harmonized, and processed using FreeSurfer (version 5.3.0). Children with excessive motion, poor scan quality, or incidental findings were excluded **(**Fig. 1**)**. This study specifically focused on cortical thickness, derived from the Desikan-Killiany-Tourville atlas, and subcortical volumes from the ASEG atlas, as described elsewhere (eMethods in Appendix 1**)**.

We included brain hubs corresponding to primary-sensory (i.e., sensory, visual, and limbic) and high-orders (default mode, salience, executive control, and attention) networks, which were selected prior as outcomes of interest and pre-registered (Appendix 2**)** (Nivins et al., 2024). These networks were selected as they are known to be disrupted during neonatal period and childhood in preterm birth (Sa de Almeida et al., 2021), and might contribute to low cognitive performance. The brain hubs corresponding to these networks are provided as eMethods in Appendix 1. Individual brain hubs from the left and right hemispheres were combined and analyzed as bilateral regions.

### 2.2 Group categorization

Children underwent neurocognitive testing using the National Institutes of Health Toolbox at 9–10 years. We used the age-adjusted total cognition composite scores (mean = 100, SD = 15) (Bleck et al., 2013), which integrates both fluid and crystallized intelligence, providing a comprehensive assessment of cognitive performance. For our analysis, children with low cognitive performance within the normal range (total cognition composite scores between < -1 SD and > -2 SD) at 9–10 years were classified as having ‘low cognitive performance’, while those with total cognition composite scores of 85 or higher (≥-1 SD) at 9–10 years were considered to have typical cognitive performance (i.e., controls). Children with total cognition composite scores of 70 or lower (≤-2 SD) were excluded **(**Fig. 1**)**.

### 2.3 Measures

The Short-Social Responsive Scale (SSRS) is a parent-reported, shortened version (11-items) of the 65-item SRS questionnaire used for screening ASD traits (Constantino, 2021). SSRS has been validated in the ABCD study (Murray et al., 2011). Each item is rated on a scale from 0 to 3, with the scores summed to yield a total score. Higher values indicate greater ASD traits.

ADHD symptoms were assessed using ADHD CBCL subscale, defined by experts based on the DSM-5 criteria from parent-reported Child Behaviour Checklist (CBCL) (Achenbach, 1991). Higher scores on this scale indicate more severe ADHD symptoms.

Both these symptom measures were based on the follow-up data at 12 years.

### 2.4 Exposures

Consistent with our earlier study (Nivins et al., 2025), gestational age was categorized into *very-preterm* (28–31 weeks), *moderately-preterm* (32–33 weeks), and *full-term* (≥39 weeks). Gestational age was retrieved from Developmental History Questionnaire survey completed by parents/caregivers.

### 2.5 Statistical analysis

All analyses conducted in this study were pre-registered unless otherwise specified (Nivins et al., 2024).

Discrete variables are reported as counts (%) and continuous variables as means (SDs). We compared demographics variables between children with low cognitive performance (total cognition composite scores: <-1 SD and > -2 SD) and those with typical cognitive performance (total cognition composite scores: ≥-1 SD) separately for very-preterm, moderately-preterm, and full-term groups, using unpaired *t*-test or χ2 test, as appropriate.

For conventional ROI analysis, primary contrast compared very-preterm children with low cognitive performance to their typical performing very-preterm peers, and moderately-preterm children with low cognitive performance to their typical performing moderately-preterm peers. The secondary contrast compared preterm children with low cognitive performance to full-term peers with typical cognitive performance, analyzed separately for both very-preterm and moderately-preterm groups. All contrasts were performed according to the pre-registered analysis plan. Mixed-effects modelling was used to test these contrasts, adjusting for age at the MRI-scan, sex assigned at birth, and socioeconomic status (SES), i.e., maternal education, household income, and neighbourhood area index as fixed effects, with scanner site included as random effect. These covariates were selected based on their established associations with brain structure in prior research (Fig. A1; eMethods in Appendix 1**)**. Models with subcortical outcomes were additionally adjusted for total brain volume. Given the novelty of this study, we did not adjust for multiplicity for ROI analysis, as doing so might increase risk of overlooking subtle but potentially clinically meaningful associations. Results are reported as standardized β values with 95 %CIs. We also tested for an interaction between group and SES (group x SES) in a separate model to assess whether SES moderates the observed differences, adjusting for aforementioned covariates. All tests were two-sided, and p ≤ 0.05 was considered statistically significant.

A sensitivity analysis was conducted by excluding children with neonatal complications (yes/no) or those as classified as having poor intrauterine group (defined as birthweight-z score < 2SD).

SCN analyses were conducted using same group contrasts as those specified for ROI analyses. Brain hubs associated with primary-sensory and high-order networks were selected. To mitigate scanner site effects on cortical thickness (Fortin et al., 2018), we applied ComBat harmonization on brain hubs (Johnson et al., 2007). Structural covariance was computed using partial correlations between harmonized brain hubs, adjusting for same covariates as above without including scanner site, resulting in 26 × 26 connectivity matrix per group. The values were then transformed into *z*-scores using Fisher’s transformation, and group differences were assessed using non-parametric permutation testing (1000 iterations), with false discovery rate (FDR) correction (p ≤ 0.05).

To measure IDSCN among significant connections in the above analysis, we extracted IDSCN values using a method proposed by Liu (Liu et al., 2021). IDSCN values represent each child’s contribution to their group’s overall structural covariance, i.e., measure of inter-regional association strength of that child. We examined association between IDSCN values and ASD/ADHD symptoms **(**eMethods in Appendix 1**)**.

Besides pre-registered analyses (Nivins et al., 2024), we conducted *post-hoc* analyses to examine whether neural patterns in very-preterm and moderately-preterm children with low cognitive performance resemble those in full-term peers with low cognitive performance. This question is clinically relevant, as similarities could suggest the potential for a one-size-fits-all intervention across gestational groups, whereas differences may highlight the need for tailored approaches. We examined previously defined brain hubs, comparing full-term children with low cognitive performance to typical performing full-term peers. Mixed-effects models were used, adjusting for the same confounders, without correction for multiplicity **(**eMethods in Appendix 1**)**.

Second, we observed stronger structural covariance among moderately-preterm children with low cognitive performance compared to their typically performing moderately-preterm peers. Given that preterm children often show altered lateralization (Lee et al., 2021), it is unclear whether these stronger connections are present in both hemispheres or more confined to one. To address this, we conducted SCN analyses within and between hemispheres, comparing very- and moderately-preterm children with low cognitive performance to their typically performing peers and to full-term peers. All analyses were corrected for multiplicity (eMethods in Appendix 1**)**.

Lastly, to interpret structural covariance patterns more objectively, we applied graph-based metrics, which provide quantitative measures of network organization and identify hub regions that play a central role in communication and integration across the networks. Analyses compared children with low cognitive performance to their typically performing peers within very- and moderately-preterm groups, as well as to full-term peers. This approach allows us to move beyond single-region comparisons and capture structural patterns that may underlie low cognitive performance in subsets of children **(**eMethods in Appendix 1**)**.

## 3 Results

### 3.1 Cohort description

A total of 7281 children met inclusion criteria for the study at 9–10 years (3802 Boys [52.2 %]; mean [SD] age, 9.9 [0.6]; 1187 [16.3 %] Black, 1570 [21.6 %] Hispanic, and 3791 [52.1 %] White participants). Among them, 71 were born very-preterm, 151 moderately-preterm, and 7056 full-term. As expected, proportion of children performing in low cognitive performance range in cognitive assessments was highest among those born very-preterm (29.58 %), followed by moderately-preterm (24.03 %), and lowest in full-term children (16.20 %).

Within very-preterm group, children with low cognitive performance were born at lower gestational age compared to their typical performing peers **(**Table 1**)**. They were taller, slightly heavier at 9–10 years, with smaller total brain volumes. They also came from lower socioeconomic backgrounds, though pregnancy-related complications were similar between groups. Similar characteristics were observed among moderately-preterm and full-term groups.

**Table 1:** Demographics.

Characteristics	Very preterm (n = 71)		Moderately preterm (n = 154)		Full-term (n = 7056)	
	Low cognitive performance (n = 21)	Typical performance (n = 50)	Low cognitive performance (n = 37)	Typical performance (n = 117)	Low cognitive performance (n = 1143)	Typical performance (n = 5913)
Gestational age, weeks	28.5 (1.7) ^***^	29.6 (1.5)	32.3 (0.4) ^***^	32.4 (0.5)	40.0 (0.08) ^***^	40.0 (0.08)
Birthweight, kg	1.6 (0.5)	1.4 (0.3)	1.7 (0.3)	1.6 (0.4)	3.1 (0.5) ^***^	3.2 (0.5)
Sex: female (%)/ male (%)	9 (12.7)/ 12 (16.9)	20 (28.2)/30 (42.3)	21 (13.6)/ 16 (10.4)	52 (33.8)/ 65 (42.2)	557 (7.9)/ 586 (8.3)	2817 (39.9)/ 3093 (43.8)
Twins	10 (14.1)	23 (32.4)	12 (7.8) ^***^	73 (47.4)	60 (0.8)	360 (5.1)
Pregnancy complications**^†^**						
Diabetes	5 (7.0)	6 (8.5)	4 (2.6)	16 (10.4)	74 (1.1) ^***^	367 (5.2)
Pre-eclampsia	5 (7.0)	12 (16.9)	11 (7.1)	31 (20.1)	57 (0.8) ^***^	263 (3.7)
Prenatal exposure						
Alcohol	1 (1.4)	1 (1.4)	1 (0.6)	4 (2.6)	22 (0.3) ^***^	175 (2.5)
Smoking	1 (1.4)	1 (1.4)	5 (3.3)	4 (2.6)	96 (1.4) ^***^	242 (3.4)
**9**–**10 years**						
Age at exam, years	9.9 (0.6) ^***^	10.0 (0.7)	9.8 (0.6) ^***^	10.0 (0.6)	9.8 (0.6) ^***^	9.9 (0.6)
Height, cm	140 (8.2) ^***^	139 (7.7)	141 (6.9) ^***^	141 (8.5)	140 (8.7) ^***^	140 (8.4)
Weight, Kg	38.0 (8.9) ^***^	36.4 (9.7)	38.9 (11.3) ^***^	37.3 (10.8)	39.4 (12.4) ^***^	37.0 (10.1)
Total brain volumes at 9–10 years	1,129,874 ^***^ (122999)	1,213,932 (123046)	1,167,939 ^***^ (132246)	1,219,820 (114304)	1,178,830 ^***^ (107077)	1,234,995 (110928)
Socioeconomic status	−1.3 (1.2) ^***^	−0.10 (1.41)	−0.70 (1.4) ^**^	0.06 (1.3)	−0.96 (1.2) ^***^	0.24 (1.3)

Data are presented as mean (SD) or n (%) unless otherwise specified. ^†^Pregnancy complications, diabetes: Very-preterm missing for 1 (typical: 1); Moderately-preterm missing for 5 (low:3, typical: 2); and Full-term missing for 217 (low:55, typical: 162); pre-eclampsia: Very-preterm missing for 1 (typical: 1); Moderately-preterm missing for 3 (low: 2, typical: 1); and Full-term missing for 231 (low: 58, typical: 173). Low cognitive performance is defined as total cognition composite scores < –1 SD and > –2 SD. Typical performance is defined as total cognition composite scores: ≥-1 SD.

### 3.2 Conventional ROI analysis

#### 3.2.1 Brain differences in low cognitive vs. Typical performing preterm

**Fig. 2:**
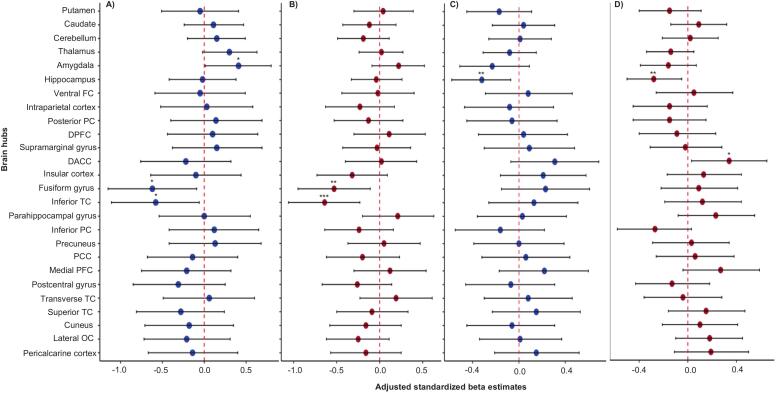
Forest plot comparing brain hubs at 9–10 years between **(A)** very-preterm children with low cognitive performance and very-preterm children with typical cognitive performance, **(B)** very-preterm children with low cognitive performance and full-term children with typical cognitive performance, **(C)** moderately-preterm children with low cognitive performance and moderately-preterm children with typical cognitive performance, and **(D)** moderately-preterm children with low cognitive performance and full-term children with typical cognitive performance. Low cognitive performance is defined as total cognition composite scores < –1 SD and > –2 SD. Typical performance is defined as total cognition composite scores: ≥-1 SD. Black lines represent confidence intervals (CIs), while blue and red dots indicate adjusted standardized beta estimates for each comparison. Abbreviations: FC, frontal cortex; PC, parietal cortex; DLFC, dorsolateral frontal cortex; DACC, dorsal anterior cingulate cortex; TC, temporal cortex; PCC, posterior cingulate cortex; PFC, prefrontal cortex; and OC, occipital cortex. *p < 0.05; **p < 0.01; ***p < 0.001.

Very-preterm children with low cognitive performance had thinner inferior temporal cortex (β = -0.58 [95 %CI, −1.11 to −0.06]; p = 0.036) and thinner fusiform gyrus (β = -0.62 [-1.15 to −0.09]; p = 0.02), but larger amygdala volumes (β = 0.41 [0.01 to 0.80]; p = 0.05) compared to typical performing peers **(**Fig. 2**A;** Table A.1**)**. Moderately-preterm children with low cognitive performance had smaller hippocampal volumes (β = -0.32 [-0.58 to −0.07]; p = 0.01) **(**Fig. 2**C)**. No other differences were observed in either group.

#### 3.2.2 Brain differences in low cognitive preterm vs. Typical performing full-term

Very-preterm children with low cognitive performance had thinner inferior temporal cortex (β = -0.64 [-1.06 to −0.23]; p=<0.001) and thinner fusiform gyrus (β = -0.53 [-0.95 to −0.11]; p = 0.01) compared to typically performing full-term peers **(**Fig. 2**B;** Table A.1**)**. Moderately-preterm children with low cognitive performance had thicker dorsal anterior-cingulate cortex (β = 0.34 [0.03 to 0.65]; p = 0.03) and smaller hippocampus volumes (β = -0.28 [-0.50 to −0.05]; p = 0.02) compared to typical performing full-term peers **(**Fig. 2**D)**. The mean and SD of all brain hubs for preterm and full-term groups are presented in Table A.2.

### 3.3 Structural covariance analysis

#### 3.3.1 Low cognitive vs. typical performing preterm

**Fig. 3:**
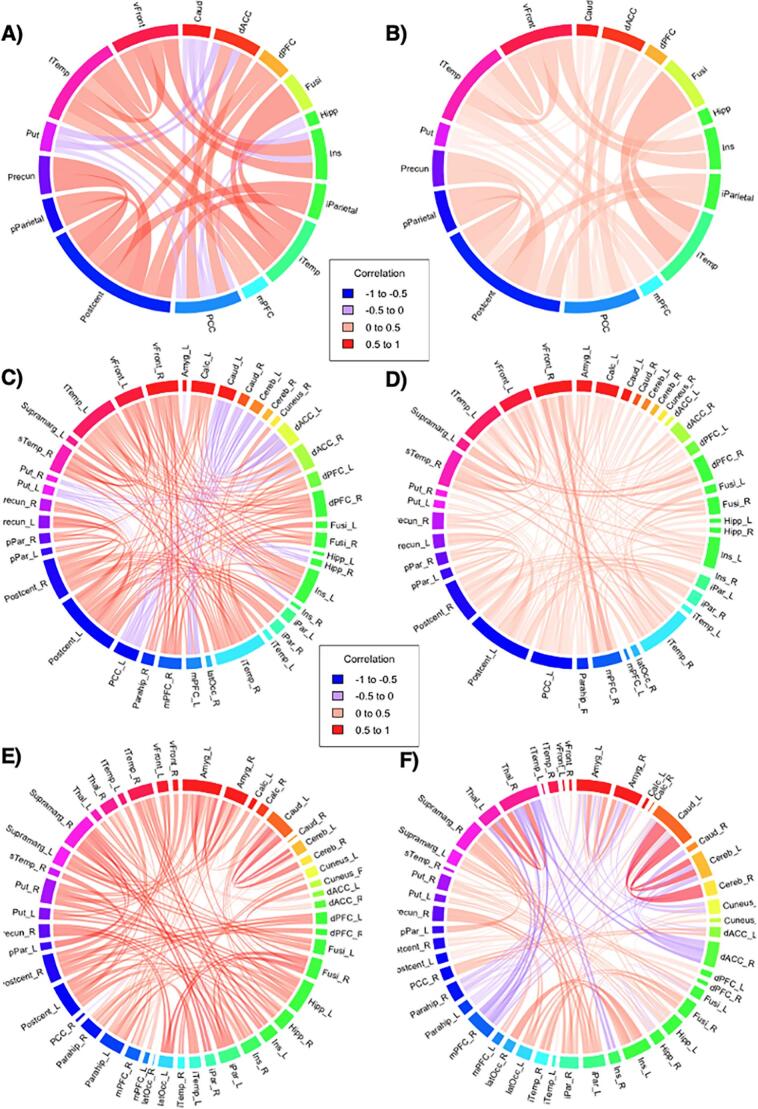
The chord diagrams illustrate structural covariance patterns across brain hubs in children aged 9–10 years. Each colored segment represents a brain hub, and the chords show significant structural correlations, with thickness and color intensity indicating the strength of these correlations. **Top left**–**right**: Moderately-preterm children with low cognitive performance **(A)** exhibited stronger structural covariance compared to moderately-preterm children with typical performance **(B). Middle left**–**right:** Between-hemispheric covariance in moderately-preterm children with low cognitive performance **(C)** had stronger compared to moderately-preterm children with typical performance **(D) Bottom left**–**right**: Between-hemispheric covariance in very-preterm children with low cognitive performance **(E)** had stronger compared to very-preterm children with typical performance **(F)**. Low cognitive performance is defined as total cognition composite scores < –1 SD and > –2 SD. Typical performance is defined as total cognition composite scores: ≥-1 SD. All associations with p-values below 0.05 are presented (uncorrected). Abbreviations: Supramarg (Supramarginal Gyrus), Calc (Calcarine), Precun (Precuneus), latOcc (Lateral Occipital), Fusi (Fusiform), Amyg (Amygdala), iTemp (Inferior Temporal), Postcent (Postcentral Gyrus), tTemp (Transverse Temporal), Ins (Insula), dPFC (Dorsal Prefrontal Cortex), sTemp (Superior Temporal), mPFC (Medial Prefrontal Cortex), PCC (Posterior Cingulate Cortex), iPar (Inferior Parietal), Parahip (Parahippocampal Gyrus), Hipp (Hippocampus), Thal (Thalamus), dACC (Dorsal Anterior Cingulate Cortex), pPar (Posterior Parietal Cortex), vFront (Ventral Frontal Cortex), Put (Putamen), Caud (Caudate Nucleus), Cereb (Cerebellum).

Very-preterm children with low cognitive performance generally showed stronger covariance between brain hub pairs compared to typical performing peers, but none survived multiple comparison correction **(**Fig. A.3**)**. Moderately-preterm children with low cognitive performance also had stronger covariance, only precuneus-postcentral gyrus pair remained significant (low cognitive: r = 0.80 vs. typical: r = 0.35) **(**Fig. 3**A to B;** Fig. A.4**)**.

#### 3.3.2 Low cognitive preterm vs. typical performing full-term

Both very-and moderately-preterm children with low cognitive performance had stronger structural covariance than typically performing full-term peers, though differences did not survive multiple comparison correction **(**Fig. A.5**)**.

### 3.4 Relationship between IDSCN and neurobehavioral scores

Higher IDSCN values for the precuneus-postcentral gyrus pair showed non-significant positive associations with ASD (R^2^ = 0.13, p = 0.45) and ADHD (R^2^ = 0.06, p = 0.74).

### 3.5 Sensitivity analysis

Findings remained consistent across all analyses. Socioeconomic status moderated the relationship between cognitive outcomes and brain measures, mainly for thalamus volumes in moderately-preterm children, with a stronger effect in low cognitive performance (β = -0.25, p = 0.01) than in typical performers (β = 0.03, p = 0.56) **(**Table A.3 to A.5**)**.

### 3.6 Post-hoc analysis

#### 3.6.1 Brain differences in low cognitive vs. Typical performing full-term

Compared to typically performing full-term peers, children with low cognitive performance had thinner cortical regions, including the pericalcarine, lateral occipital, cuneus, postcentral, inferior parietal, parahippocampal, inferior temporal, fusiform, supramarginal, posterior parietal, and intraparietal cortices (β = -0.08 to −0.17), and thicker medial prefrontal cortex **(**Fig. A.2; Table A.6**)**. They also had smaller hippocampal, caudate, and cerebellar volumes.

#### 3.6.2 Within- and between-hemisphere structure connection

In the very-preterm group, within-hemisphere analysis revealed no differences **(**Fig. A.6A to A.6D**)**, whereas between-hemisphere analyses indicated stronger covariance in children with low cognitive performance for the left inferior temporal-left parahippocampal gyrus pair (compared to typical-performing very-preterm peers) and the left insular-left postcentral gyrus pair (compared to typical-performing full-term peers) **(**Fig. 3**E to F)**.

In the moderately-preterm group, within-hemisphere analysis revealed stronger covariance in children with low cognitive performance for left precuneus-left postcentral gyrus pair **(**Fig. A.6E to A.6H**)**, whereas between-hemisphere analyses showed stronger covariance in the left precuneus-left postcentral gyrus, left ventral frontal-left insula, and right superior temporal-left ventral frontal pairs **(**Fig. 3**C to D)**. When compared with typical performing full-term children, moderately-preterm children with low cognitive performance exhibited stronger within-hemisphere covariance in the right dorsal prefrontal-right parahippocampal pair and stronger between-hemisphere covariance in the left ventral frontal-right superior temporal pair **(**Fig. A.7**)**. Results not surviving multiple comparison are reported in Supplementary material **(**Fig. A.6 to A.7**).**

#### 3.6.3 Graph-theory results

**Fig. 4:**
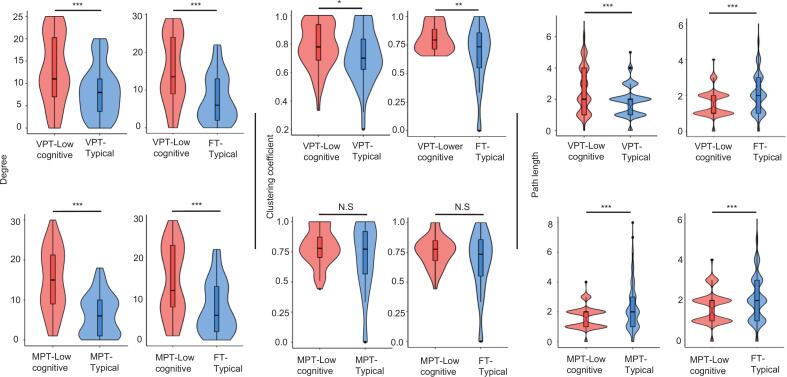
Comparison of graph measures (degree, clustering coefficient, and path length) between the low cognitive performance group and typical performance group in very preterm **(top row)** and moderately preterm **(bottom row)** children aged 9–10 years. Violin plots illustrate the distribution of graph measures for each group, with fill colors indicating cognitive performance status (low cognitive: orange; typical: blue). Boxplots overlaid on the violins show the median, interquartile range, and outliers for each group. Low cognitive performance is defined as total cognition composite scores < –1 SD and > –2 SD. Typical performance is defined as total cognition composite scores: ≥-1 SD.Abbreviations: VPT-Lower cognitive, Very-preterm children with low cognitive performance; VPT-Typical, Very-preterm children with typical cognitive performance; MPT- Lower cognitive, Moderately-preterm children with low cognitive performance; MPT-Typical, Moderately-preterm children with typical cognitive performance; FT-Typical, Full-term children with typical cognitive performance. *p < 0.05; **p < 0.01; ***p < 0.001; N.S, non-significant (For interpretation of the references to color in this figure legend, the reader is referred to the web version of this article.).

In the very-preterm group, children with low cognitive performance showed higher degree and clustering coefficient, as well as longer path length compared to typically performing very-preterm peers. Compared to typically performing full-term peers, these children exhibited higher degree and clustering coefficient but shorter path length **(**Fig. 4**)**.

**Fig. 5:**
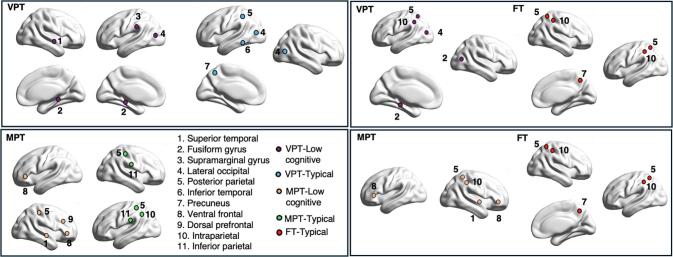
Brain-hubs for very-preterm and moderately-preterm children at 9–10 years of age. The figure shows representative brain regions that act as central hubs for cognitive processing in children across different gestational groups. Comparisons are made between children with low cognitive performance and their peers with typical performance, both within the same preterm groups and with full-term peers, illustrating distinct hub distributions and pattern. **Top-left:** VPT children with low cognitive performance vs. VPT children with typical performance; **Top-right:** VPT children with low cognitive performance vs. FT children with typical performance. **Bottom-left:** MPT children with low cognitive performance vs. MPT children with typical performance; **Bottom-right:** MPT children with low cognitive performance vs. FT children with typical performance. Low cognitive performance is defined as total cognition composite scores < –1 SD and > –2 SD. Typical performance is defined as total cognition composite scores: ≥-1 SD. Abbreviations: VPT-Low cognitive, Very-preterm children with low cognitive performance; VPT-Typical, Very-preterm children with typical cognitive performance; MPT-Low cognitive, Moderately-preterm children with low cognitive performance; MPT-Typical, Moderately-preterm children with typical cognitive performance; FT-Typical, Full-term children with typical cognitive performance.

In the moderately-preterm group, children with low cognitive performance demonstrated higher degree and shorter path length compared to both typically performing moderately-preterm and full-term peers, with no significant differences in clustering coefficient **(**Fig. 4**)**. Brain-hubs for each contrast are shown in **(**Fig. 5**).**

## 4 Discussion

This study, for the first time, leveraged two methodologies to investigate neural mechanisms underlying cognitive outcomes variability among very and moderately-preterm children. Our findings show that the neural mechanism associated with low cognitive performance at 9–10 years differs by degree of prematurity. Very-preterm children exhibited thinner inferior temporal and thinner fusiform gyrus and larger amygdala volumes, while moderately-preterm children had smaller hippocampal volumes. Importantly, these patterns differed from those seen in full-term children with low cognitive performance, who showed wide-spread alterations, including in prefrontal and intraparietal sulcus, suggesting distinct neural architecture across gestational ages. Preterm children with low cognitive performance also exhibited stronger structural covariance between brain hub pairs; however, after correcting for multiple comparisons, these effects were significant only in the moderately-preterm group. *Post-hoc* graph-theory analyses supported these findings, showing higher degree and clustering coefficient, and shorter path length among brain hubs. Moreover, preterm children with low cognitive performance exhibited altered hub topology compared with typically performing peers across both preterm and full-term groups, highlighting how differences in hub involvement may contribute to heterogeneity of cognitive outcomes following preterm birth.

Very-preterm children with low cognitive performance had thinner inferior temporal cortex, fusiform gyrus, and larger amygdala volumes compared to both typical-performing very-preterm and full-term peers, consistent with previous studies indicating vulnerabilities in these regions (Mueller et al., 2022, Nosarti et al., 2008). These structural alterations are particularly significant given the critical roles of inferior temporal and fusiform gyrus in higher-order visual processing, including object recognition, face perception, and visual memory (Conway, 2018, Weiner and Zilles, 2016). Particularly, the inferior temporal cortex integrates complex visual information and connects it with semantic knowledge, while the fusiform gyrus is crucial for face recognition and visual word processing (Zhang et al., 2016). The amygdala, on the other hand, is connected to higher-order visual processing areas and plays a central role in processing emotional and social stimuli and can influence visual-spatial processing through its role in attention and memory for emotionally salient stimuli. Alterations in these structures, including the amygdala, could contribute to difficulties in visual-spatial processing, as we have previously shown (Bolk et al., 2018a, Bolk et al., 2018b), potentially explaining why some children perform below their peers.

We found that moderately-preterm children with low cognitive performance had smaller hippocampal volumes at 9–10 years compared to those without, along with a pattern of thicker dorsal anterior cingulate cortex when compared to typically developing full-term children. This finding is novel, as existing studies on moderately-preterm birth have mainly focused on functional developmental outcomes, with limited attention to neural measures beyond neonatal or early childhood periods (Munakata et al., 2013, Rogers et al., 2014, Thompson et al., 2008, Walsh et al., 2014). Although few studies have reported smaller brain volumes, including the hippocampus (Alnæs et al., 2020, Meng et al., 2016, Nath et al., 2023, Peterson et al., 2000), direct comparisons with our findings are challenging. Still, our results contribute to understanding why some moderately-preterm children experience cognitive difficulties, while others not. The hippocampus, crucial for spatial navigation, planning, recall, and working memory tasks, serves as a neural hub for integrating spatial, temporal, and associative information (Beauchamp et al., 2008, Buzsáki and Moser, 2013), which may explain cognitive challenges seen in moderately-preterm children. The observed thicker dorsal anterior cingulate cortex may reflect a maturational delay, and as part of salience network, it plays an important role in regulating attention and cognitive control (Yerys et al., 2019), which could also explain the cognitive challenges faced by these children.

Consistent with our hypothesis, we found that low cognitive performances were associated with distinct patterns of brain involvement across degrees of prematurity, as discussed above. This distinct involvement likely reflects differential maturational vulnerabilities associated with prematurity (Inder et al., 2023, Sannia et al., 2015). Further, these children are subjected to different environmental stressors during the neonatal period, e.g., NICU, which could also be associated with these distinct patterns (Smith et al., 2011). Interestingly, these patterns differ from full-term children with low cognitive performance, who, in a *post-hoc* comparison showed widespread involvement across multiple brain regions, which aligns with previous studies showing associations between intelligence and discrete brain regions such as prefrontal, parietal regions (Choi et al., 2008, Karama et al., 2009, Luders et al., 2009, Menary et al., 2013, Narr et al., 2007).

Besides the distinct pattern observed in the ROI analysis, moderately-preterm children with low cognitive performance exhibited stronger structural covariance in precuneus-postcentral gyrus pair compared to typically performing moderately-preterm peers. Importantly, both regions are part of parietal cortex, and are anatomically and functionally interconnected. The postcentral gyrus, as primary somatosensory cortex, processes tactile and proprioceptive information, whereas precuneus, part of default-mode network, is involved in visuospatial imagery, self-related processing, and aspects of consciousness. These regions are connected by white matter tracts including the superior longitudinal fasciculus and short association fibers and co-activation has been observed in tasks involving body-awareness, visuospatial-integration, and motor-imagery, suggesting functional interaction (Margulies et al., 2009). This finding was robust, as it remained consistent across subsequent within-and between-hemisphere analyses. This is further supported through our *post-hoc* graph theory metrics analysis which indicated higher degree and shorter path-length and trend for higher clustering coefficient among moderately-preterm children with low cognitive performance, which is consistent with previous studies on preterm children, which have reported altered topology of structural networks (Gozdas et al., 2018, Zhao et al., 2019, Zheng et al., 2023). Our observation of stronger structural covariance between precuneus-postcentral gyrus likely reflect a compensatory mechanism in response to *in-utero* disruption during the third trimester, a critical phase for cortical development marked by rapid synaptogenesis and dendritic arborization. This compensatory mechanism may involve the reorganization of brain hubs, as supported by the presence of different hubs for cognitive processing compared to typically performing moderately preterm peers.

Multiple studies have speculated that structural disruptions following preterm-birth may be associated with higher rates of neurodevelopmental disorders, e.g., ADHD. In this study, we took a step further by extracting IDSCN values from the disrupted precuneus-postcentral gyrus pair, as discussed earlier, and examined their potential association with the ASD/ADHD symptoms. We did not observe a significant association, which may, in part be due to the limited sample size.

From a clinical perspective, an important question remains, ‘Why some preterm children perform within normal cognitive range while others do not?’. In this study, children with typical cognitive performance had lower degree, lower clustering coefficient, and distinct central hubs compared to those with cognitive problems, suggesting that factors, such as lesser neonatal complications or more enriched environmental experiences, might likely contribute to better outcomes.

The study had several strengths. A pre-registered analysis plan was implemented to mitigate risk of false-positive results, ensuring robustness and transparency. The study was hypothesis-driven involving large cohort of children (>7000) which allowed us to detect small effect-sizes. To categorize groups, a full-scale cognitive composite-score was used rather than specific-tests. The analysis part combined both ROI-and network-based analyses instead of conventional methods, offering a broader perspective on brain-structure and function. Unlike prior studies that focus on group-level measures, IDSCN values were extracted for each-child with difficulties and correlated with symptom measures. To minimize variability, site effects were harmonized using ComBat prior to network analysis (Fortin et al., 2017). Further, none of the preterm-children had focal brain lesions at 9–10 years, making the sample more representative of most survivors of modern neonatal practice.

## 5 Limitations

Several limitations should be considered in this study. ROI analyses were not corrected for multiple comparisons, and multiple *post-hoc* analyses (e.g., within- and between hemisphere SCN analysis, graph metrics) were conducted that were not pre-registered. These findings should therefore be interpreted with caution due to increased risk of type 1 error, although they provide novel information. Analysis focused only on cortical thickness and subcortical volumes, subsequent studies should consider other brain metrics (e.g., gyrification indices). Gestational age was based on parent-report questionnaires, which are subject to recall-bias. However, studies have shown that maternal recall of neonatal variables up to nine years post-birth aligns with medical records (Rice et al., 2007). We lack data on neonatal complications, which limits our ability to fully account for their potential influence on outcomes. Group sample sizes were uneven, which may affect the stability and generalizability of comparisons, particularly for SCN and graph-theory analyses that are sensitive to sample size and variance. Furthermore, results were based on a single dataset, future studies should validate these results using other datasets. Smoking and alcohol use were self-reported and may be subject to social desirability bias. Although smaller brain structures were observed in children with low cognitive performance, these results were based on single-time point, making it difficult to determine whether observed differences were transient or persistent. Finally, despite adjusting for known factors associated with brain outcomes, unmeasured confounders, such as genetics, may still influence the results.

## 6 Conclusions

Our results suggest that distinct neural mechanism underlying low cognitive performance in preterm-children, vary by degree of prematurity. Very preterm children exhibit altered inferior temporal and fusiform gyrus, and amygdala volumes, while moderately preterm children show altered hippocampal structures. Moderately preterm children with low cognitive performance also tend to exhibit stronger covariance within brain hubs, reflecting compensatory mechanisms in response to early-neuronal disruptions. Overall, our findings highlight the importance of tailoring interventions based on prematurity and associated neural profiles.

## 7 Funding/Support

This study was supported by the Stiftelsen Frimurare Barnhuset, Stockholm and TORE NILSONS STIFTELSE FÖR MEDICINSK FORSKNING through postdoctoral grant C41160433 and K62408423 (Dr Nivins). This study was also supported by grant FO2023-0178 from the Swedish Brain Foundation and grant 2023–02451 from the Swedish Medical Research Council (Dr Ådén) and Stiftelsen Frimurare Barnhuset, Stockholm (Dr Padilla). The funders had no role in the design and conduct of the study; collection, management, analysis, and interpretation of the data; preparation, review, or approval of the manuscript; and decision to submit the manuscript for publication.

## CRediT authorship contribution statement

**Samson Nivins:** Writing – original draft, Visualization, Methodology, Investigation, Formal analysis, Conceptualization. **Nelly Padilla:** Writing – review & editing, Validation, Methodology, Conceptualization. **Hedvig Kvanta:** Writing – review & editing. **Gustaf Mårtensson:** Writing – review & editing. **Ulrika Ådén:** Writing – review & editing, Supervision, Methodology, Investigation, Funding acquisition, Conceptualization.

## Declaration of Competing Interest

The authors declare that they have no known competing financial interests or personal relationships that could have appeared to influence the work reported in this paper.

## Data Availability

Data will be made available on request.
