# Supplementary material for: Distinct neural mechanisms underlying cognitive difficulties in preterm children born at different stages of prematurity
Source: Neuroimage Clin. 2025 Sep 3;48:103876. doi: 10.1016/j.nicl.2025.103876 (PMC12454289; doi:10.1016/j.nicl.2025.103876)
Supplement: Supplementary Data 1 [file mmc1.docx]

**Supplementary Material 1**

**Distinct neural mechanisms underlying cognitive difficulties in preterm children born at different stages of prematurity**

Samson Nivins PhD**^*^**, Nelly Padilla PhD, Hedvig Kvanta PhD, Gustaf Mårtensson PhD, Ulirka Ådén PhD

**^*^Corresponding author:**

Dr Samson Nivins PhD

Woman and Child Health, Pediatrics,

Karolinska Institute, Stockholm, Sweden

Email: [samson.nivins@ki.se](mailto:samson.nivins@ki.se)

Phone: +46 769794725

Table of Contents

[eMethods 4](#_Toc206366396)

[MRI components 4](#_Toc206366397)

[Image acquisition 4](#_Toc206366398)

[Initial Quality Control 4](#_Toc206366399)

[Incidental Findings 4](#_Toc206366400)

[Image Pre-Processing 4](#_Toc206366401)

[Regions corresponding to brain-hubs 4](#_Toc206366402)

[Statistical analysis 5](#_Toc206366403)

[Confounder 5](#_Toc206366404)

[ComBat harmonization 5](#_Toc206366405)

[Individualized differential structural covariance network (IDSCN) 5](#_Toc206366406)

[*Post-hoc* analysis 5](#_Toc206366407)

[ROI analysis: Brain differences in low cognitive vs. typical performing full-term 5](#_Toc206366408)

[Structural Covariance Analysis 6](#_Toc206366409)

[Graph theory 6](#_Toc206366410)

[eResults 8](#_Toc206366411)

[Table A.1 Brain hubs comparison at 9–10 years of age between children with low cognitive performance and those with typical performance 8](#_Toc206366412)

[Table A.2 Mean and SD of brain hubs among very preterm, moderately preterm, and full-term children with low cognitive performance and typical performance 10](#_Toc206366413)

[Table A.3 Sensitivity analysis: Comparison of brain-hubs at 9–10 years of age between low cognitive performance and typical performance in very-preterm groups 12](#_Toc206366414)

[Table A.4 Sensitivity analysis: Comparison of brain-hubs at 9–10 years of age between low cognitive performance and typical performance in moderately-preterm groups 13](#_Toc206366415)

[Table A.5 Interaction effects of cognitive performance and socioeconomic status on brain hubs in preterm children aged 9-10 years 14](#_Toc206366416)

[Table A.6 Brain hubs comparison at 9-10 years of age between full-term children with low cognitive performance and typical performance 15](#_Toc206366417)

[eFigures 16](#_Toc206366418)

[Fig A.1 Covariates selected based on their association with brain outcomes 16](#_Toc206366419)

[Fig A.2 Forest plot comparing brain hubs between full-term children with low cognitive performance and full-term with typical performance. 17](#_Toc206366420)

[Fig A.3 Partial correlation of structural associations between brain hubs in very-preterm children grouped by cognitive performance at 9-10 years of age. 18](#_Toc206366421)

[Fig A.4 Partial correlations of structural associations between brain hubs in moderately-preterm children stratified by cognitive performance at 9-10 years of age. 19](#_Toc206366422)

[Fig A.5 Partial correlations of structural associations between brain hubs in 9-10-year-old children, stratified by cognitive performance and gestational age groups. 20](#_Toc206366423)

[Fig A.6 Chord diagrams illustrating structural covariance patterns within hemispheres in 9-10-year-old children. 21](#_Toc206366424)

[Fig A.7 Chord diagrams illustrating structural covariance patterns within and between hemispheres in 9-10-year-old children 23](#_Toc206366425)

[References 24](#_Toc206366426)

# eMethods

## MRI components

### Image acquisition

Children underwent brain scans at 9-10 years of age on three different 3.0 Tesla MRI scanners across 21 imaging sites, using a standard adult-sized head coil. The scanners included GE Discovery MR750 (General Electric Healthcare, Milwaukee, USA), Siemens Prisma (Siemens Medical Solutions, Erlangen, Germany), and Philips Ingenia CX (Philips Medical Systems). To ensure consistency across sites and scanners, a unified protocol was implemented. During the scans, children watched a child-friendly movie to help minimize movement.[1]

Three-dimensional T1-weighted structural images were acquired using a magnetization-prepared rapid acquisition gradient-echo sequence, with the following parameters: image matrix=256 × 256, repetition time=2500 ms, echo time=2.88 ms, field of view=256 × 256 mm, flip angle= 8°, slice thickness=1.0 mm, and 176 slices.[1]

### Initial Quality Control

To mitigate motion-related artifacts, real-time automated motion detection and correction were employed using software implemented by the ABCD Data Analysis, Informatics, and Resource Center. Signal-to-noise ratio and head motion statistics were automatically computed for preliminary quality control. Images were then reviewed manually by trained technicians and graded as follows: 0 (absent), 1 (mild), 2 (moderate), and 3 (severe). Based on automated and manual quality assessments, 671 children with poor image quality were excluded from further processing.[2]

### Incidental Findings

All brain MRI scans were reviewed by board-certified neuroradiologists for clinical evaluation. Findings were graded as follows: 0 (image artifacts prevent assessment), 1 (no abnormal findings), 2 (normal anatomical variant), 3 (consider referral), and 4 (consider immediate referral). Children with scores of three or higher were excluded from the study to ensure clinical safety.[3]

### Image Pre-Processing

Structural T1-weighted images were processed using FreeSurfer version 5.3.0 (Martinos Center for Biomedical Imaging, Charlestown, USA; <https://surfer.nmr.mgh.harvard.edu/>).[4] The standard pipeline included motion correction, skull stripping, intensity normalization, Talairach transformation, grey/white matter tessellation, and topology correction. For cortical reconstruction, we used cortical thickness as it provides a sensitive measure of cortical maturation and potential structural alterations. The parcellation of the cortex was performed using the Desikan-Killiany atlas,[5] which was also used to calculate total brain volumes. The ASEG atlas from FreeSurfer was used to segment subcortical structures.[1, 2, 6, 7]

Post-processing quality control of the segmented images was also performed by the ABCD team and graded as follows: 0 (absent), 1 (mild), 2 (moderate), and 3 (severe). The structural images were deemed poorly segmented or incomplete and were excluded from the analysis.

## Regions corresponding to brain-hubs

The sensory network included the postcentral gyrus, transverse temporal cortex, superior temporal cortex, and insula cortex. The visual network comprised the pericalcarine cortex, lateral occipital cortex, cuneus, and fusiform gyrus. The limbic network consisted of the parahippocampal gyrus, hippocampus, amygdala, and thalamus.

The salience network included the dorsal anterior cingulate cortex, with the insula cortex also contributing but primarily categorized under the sensory network. The dorsal attention network consisted of the intraparietal cortex and posterior parietal cortex. The default mode network included the medial prefrontal cortex, posterior cingulate cortex, and precuneus. The frontoparietal network comprised the inferior parietal cortex, dorsal prefrontal cortex, and ventral frontal cortex. Further, the cerebellum, caudate, and putamen were considered due to their broad involvement across multiple networks.

## Statistical analysis

### Confounder

The covariates were selected based on the association with the outcomes based on previous research **(eFigure 1)**.[8-10]

### ComBat harmonization

We opted for ComBat harmonisation instead of using scanner-site as a random effect as recommended by the ABCD team because including scanner-site as a random effect in structural covariance network (SCN) analysis complicates the interpretation of partial correlations. By adjusting for scanner-site variability through ComBat, we reduced confounding from scanner-site differences while focusing on the core structural relationships of interest. This approach not only preserves the robustness of the analysis but also improves statistical power by reducing unnecessary variance from site effects. Further, it ensures better comparability across sites, making the findings more generalisable.

### Individualized differential structural covariance network (IDSCN)

To compute the IDSCN, we applied the method proposed by Liu.[11] The following steps were performed: First, we constructed the reference structural covariance network (rSCN) using the moderately-preterm children with typical cognitive performance (defined as (≥-1 SD) on composite cognitive scores using the NIH Toolbox). We calculated partial correlations between brain hubs (i.e., precuneus and postcentral gyrus pair), adjusting for age, sex, and socioeconomic status. The precuneus and postcentral gyrus pair was selected because it showed significant differences that survived multiple comparison in our previous analysis.

Next, each child from the low cognitive performance group (defined as <–1 SD and >–2 SD on composite cognitive scores using the NIH Toolbox) was added to the typical performance group. We then created a new perturbed structural covariance network (pSCN) by calculating partial correlations for the same brain region pair (i.e., precuneus and postcentral gyrus)

We then calculated the difference (ΔSCN) between the pSCN and rSCN as follows:

ΔSCN = pSCN − rSCN.

The Z-score for ΔSCN was calculated using the formula:

*Z = ΔSCN/ Square root of (1 - r^2^)/ (n - 2)*

Where r represents the partial correlation from the rSCN, and n is the sample size of the combined group (i.e., children with low cognitive performance and typical performers). The Z-scores reflect the strength of structural covariance differences between each child with low cognitive performance and the reference group (i.e., typical performers)

Finally, the resulting IDSCN values were correlated with clinical symptom scores (i.e., ASD/ADHD) to investigate the relationship between individualized structural covariance alterations and symptom scores.

## *Post-hoc* analysis

### ROI analysis: Brain differences in low cognitive vs. typical performing full-term

Beyond our pre-registered analyses,[12] we conducted exploratory *post-hoc* analyses to further characterize neural patterns associated with cognitive problems.

In particular, we investigated whether the neural patterns seen in very-preterm and moderately-preterm children with low cognitive performance resemble those of full-term peers with low cognitive performance. Understanding these similarities or differences may inform whether interventions could be generalized across gestational groups or need to be tailored.

To address this, we focused on previously defined brain hubs implicated in both primary-sensory and higher-order association networks. Full-term children with low cognitive performance (total cognition composite scores <–1 SD and >–2 SD) were compared with full-term children with typical cognitive performance (scores ≥ -1 SD). We applied mixed-effects modeling to evaluate this contrast, adjusting for potential confounders: age at MRI scan, sex assigned at birth, and a composite measure of socioeconomic status (SES; maternal education, household income, and neighbourhood area index) as fixed effects. Scanner site was included as a random effect to account for potential site-specific variability.

This approach allowed us to systematically examine whether low cognitive performance in full-term children is associated with alterations in brain hubs, complementing the primary analyses of preterm groups and providing a more detailed characterization of network-level patterns relevant to cognitive function.

### Structural Covariance Analysis

Separating intra- and inter-hemispheric analyses is particularly relevant for preterm children, who often exhibit altered lateralization patterns.[13] Examining connectivity within each hemisphere allows for detection of region-specific coordination changes, whereas inter-hemispheric analyses capture potential compensatory or disrupted communication between hemispheres. This distinction provides insight into whether structural covariation alterations in preterm children are globally distributed or hemisphere-specific, which may have implications for understanding the neurobiological basis of low cognitive performance and guiding targeted interventions.

SCN analyses were performed to assess intra- and inter-hemispheric structural covariation patterns in relation to cognitive performance. Analyses were conducted separately for each hemisphere and across hemispheres. We focused on previously defined brain hubs associated with primary sensorimotor and higher-order association networks. To account for potential scanner-related variability in cortical thickness measurements, ComBat harmonization was applied to all selected hubs prior to analysis. Structural covariance was then quantified using partial correlations between harmonized brain hubs, adjusting for age at MRI scan, sex assigned at birth, and SES, consistent with covariates used in the primary analyses; scanner site was excluded as a covariate due to harmonization.

For both within- and between-hemisphere analyses, comparisons were performed between children with low cognitive performance and their typically performing peers, separately within very-preterm and moderately-preterm groups, and between preterm children (very-preterm and moderately-preterm analyzed separately) with low cognitive performance and full-term children with typical cognitive performance.

Connectivity matrices were constructed as follows: a 26×26 matrix for within-hemisphere analyses, and a 52×52 matrix for between-hemisphere analyses. Correlation coefficients were transformed to z-scores using Fisher’s r-to-z transformation. Group differences were evaluated using non-parametric permutation testing (1000 iterations), with false discovery rate (FDR) correction applied at p<0.05 to control for multiple comparisons.

### Graph theory

To interpret structural covariance patterns more objectively, we applied graph-based metrics, which provide quantitative measures of network organization and allow identification of hub regions that play a central role in communication and integration across networks. This approach moves beyond single-region comparisons, capturing distributed structural patterns that may underlie low cognitive performance in subsets of children.

Brain structural network-connectivity was explored using graph theory in R Studio (igraph package; <https://igraph.org/>).[14] on harmonised data (ComBat harmonization as discussed above). For each contrast very-preterm children with low cognitive performance versus typically performing very-preterm peers, moderately-preterm children with low cognitive performance versus typically performing moderately-preterm peers, and preterm children with low cognitive performance versus typically performing full-term peers, we computed partial correlation matrices for each pair of brain hubs, adjusting for covariates. An absolute correlation threshold of >0.3 was applied to generate binary adjacency matrices, where edges indicated strong partial correlations between hubs. These matrices were then used to construct undirected graphs, with nodes representing brain hubs and edges reflecting significant partial correlations.

Global network properties including nodal degree, clustering coefficient, and path length were calculated for each contrast. Nodal degree refers to the number of connections a node has with other nodes. Clustering coefficient measures how well a node’s neighbours are interconnected, calculated as the ratio of actual connections among neighbours to the maximum possible connections. Path length refers to the minimum number of edges required to connect one node to another. Statistical significance between groups was assessed using the Mann-Whitney U test (α = 0.05) for each contrast.

To investigate the relative importance of each brain hub, we computed degree, betweenness, and closeness centrality scores for the same contrasts. Brain hubs were then ranked by degree centrality in descending order, and the top five hubs with the highest degree were identified and compared between children with low cognitive performance and typically performing peers within each contrast as above. This approach allowed assessment of whether important hub regions differ between groups, providing insight into the network-level organization underlying low cognitive performance.

# eResults

## Table A.1 Brain hubs comparison at 9–10 years of age between children with low cognitive performance and those with typical performance

| **Brain hubs** | **Children with low cognitive vs. Typical performance** | | | |
| --- | --- | --- | --- | --- |
|  | **VPT^1^** | **MPT^2^** | **VPT vs. FT^3^** | **MPT vs. FT^4^** |
| Pericalcarine cortex | -0.14 (-0.67 - 0.40) | 0.15 (-0.21 - 0.52) | -0.16 (-0.57 - 0.25) | 0.19 (-0.11 - 0.5) |
| lateral occipital cortex | -0.21 (-0.72 - 0.31) | 0.01 (-0.34 - 0.37) | -0.25 (-0.62 - 0.11) | 0.18 (-0.1 - 0.45) |
| Cuneus | -0.18 (-0.71 - 0.35) | -0.06 (-0.45 - 0.31) | -0.16 (-0.58 - 0.25) | 0.10 (-0.21 - 0.41) |
| Superior temporal cortex | -0.28 (-0.81 - 0.24) | 0.15 (-0.23 - 0.53) | -0.09 (-0.50 - 0.33) | 0.15 (-0.16 - 0.47) |
| Transverse temporal cortex | 0.06 (-0.49 - 0.60) | 0.08 (-0.30 - 0.46) | 0.19 (-0.23 - 0.61) | -0.04 (-0.36 - 0.28) |
| Postcentral gyrus | -0.31 (-0.85 - 0.25) | -0.07 (-0.46 - 0.31) | -0.26 (-0.67 - 0.14) | -0.13 (-0.43 - 0.18) |
| Medial prefrontal cortex | -0.21 (-0.75 - 0.32) | 0.22 (-0.17 - 0.60) | 0.12 (-0.30 - 0.54) | 0.27 (-0.04 - 0.59) |
| Posterior cingulate cortex | -0.14 (-0.68 - 0.40) | 0.06 (-0.32 - 0.44) | -0.2 (-0.62 - 0.23) | 0.06 (-0.26 - 0.38) |
| Precuneus | 0.13 (-0.42 - 0.68) | 0.001 (-0.39 - 0.39) | 0.05 (-0.37 - 0.47) | 0.03 (-0.29 - 0.34) |
| Inferior parietal cortex | 0.12 (-0.42 - 0.65) | -0.16 (-0.55 - 0.22) | -0.24 (-0.64 - 0.16) | -0.27 (-0.58 - 0.03) |
| Parahippocampal gyrus | 0 (-0.54 - 0.55) | 0.03 (-0.36 - 0.41) | 0.21 (-0.20 - 0.63) | 0.23 (-0.08 - 0.55) |
| Inferior temporal cortex | **-0.58 (-1.11 - -0.06)** | 0.13 (-0.26 - 0.51) | **-0.64 (-1.06 - -0.23)** | 0.12 (-0.19 - 0.44) |
| Fusiform gyrus | **-0.62 (-1.15 - -0.09)** | 0.23 (-0.15 - 0.61) | **-0.53 (-0.95 - -0.11)** | 0.09 (-0.22 - 0.41) |
| Insula cortex | -0.1 (-0.64 - 0.44) | 0.21 (-0.16 - 0.58) | -0.32 (-0.73 - 0.09) | 0.13 (-0.17 - 0.44) |
| Dorsal anterior cingulate cortex | -0.22 (-0.76 - 0.32) | 0.31 (-0.07 - 0.69) | 0.02 (-0.40 - 0.43) | **0.34 (0.03 - 0.65)** |
| Supramarginal gyrus | 0.15 (-0.38 - 0.69) | 0.09 (-0.30 - 0.48) | -0.03 (-0.43 - 0.36) | -0.02 (-0.31 - 0.28) |
| Dorsal prefrontal cortex | 0.1 (-0.44 - 0.64) | 0.04 (-0.35 - 0.42) | 0.11 (-0.3 - 0.53) | -0.09 (-0.40 - 0.23) |
| Posterior parietal cortex | 0.14 (-0.40 - 0.69) | -0.06 (-0.45 - 0.33) | -0.13 (-0.53 - 0.27) | -0.15 (-0.45 - 0.15) |
| Intraparietal cortex | 0.03 (-0.52 - 0.58) | -0.08 (-0.47 - 0.30) | -0.23 (-0.63 - 0.17) | -0.15 (-0.45 - 0.16) |
| Ventral frontal cortex | -0.05 (-0.59 - 0.49) | 0.08 (-0.29 - 0.46) | -0.02 (-0.44 - 0.4) | 0.05 (-0.26 - 0.37) |
| Hippocampus | -0.02 (-0.42 - 0.38) | **-0.32 (-0.58 - -0.07)** | -0.04 (-0.33 - 0.26) | **-0.28 (-0.5 - -0.05)** |
| Amygdala | 0.41 (0.01 - 0.80) | -0.23 (-0.51 - 0.09) | 0.22 (-0.09 - 0.52) | -0.16 (-0.39 - 0.07) |
| Thalamus | 0.30 (-0.02 - 0.63) | -0.08 (-0.31 - 0.15) | 0.02 (-0.24 - 0.27) | -0.14 (-0.34 - 0.05) |
| Cerebellum | 0.15 (-0.20 - 0.49) | 0.01 (-0.26 - 0.28) | -0.19 (-0.49 - 0.11) | 0.02 (-0.21 - 0.25) |
| Caudate | 0.11 (-0.24 - 0.47) | 0.04 (-0.23 - 0.31) | -0.12 (-0.43 - 0.19) | 0.09 (-0.14 - 0.32) |
| Putamen | -0.05 (-0.51 - 0.41) | -0.17 (-0.45 - 0.11) | 0.04 (-0.3 - 0.39) | -0.15 (-0.40 - 0.11) |

Data are presented as β (95%CI). Significant regions are highlighted in bold. Abbreviations: VPT, very preterm; MPT, moderately preterm; FT, full-term. ^1^VPT children with low cognitive performance vs. VPT with typical performance; ^2^MPT children with low cognitive performance vs. MPT with typical performance; ^3^VPT children with low cognitive performance vs. FT with typical performance; ^4^MPT children with low cognitive performance vs. FT with typical performance.

Low cognitive performance is defined as total cognition composite scores <–1 SD and >–2 SD. Typical performance is defined as total cognition composite scores: ≥-1 SD.

## Table A.2 Mean and SD of brain hubs among very preterm, moderately preterm, and full-term children with low cognitive performance and typical performance

| **Brain hubs** | **VPT** | | **MPT** | | **FT** | |
| --- | --- | --- | --- | --- | --- | --- |
|  | **Low cognitive** | **Typical** | **Low cognitive** | **Typical** | **Low cognitive** | **Typical** |
| Pericalcarine cortex | 1.8 (0.15) | 1.82 (0.14) | 1.85 (0.11) | 1.85 (0.14) | 1.8 (0.14) | 1.83 (0.13) |
| lateral occipital cortex | 2.31 (0.12) | 2.36 (0.14) | 2.36 (0.11) | 2.38 (0.11) | 2.31 (0.12) | 2.34 (0.12) |
| Cuneus | 2.09 (0.11) | 2.12 (0.12) | 2.12 (0.13) | 2.14 (0.12) | 2.09 (0.12) | 2.12 (0.12) |
| Superior temporal cortex | 3.04 (0.14) | 3.09 (0.15) | 3.07 (0.13) | 3.07 (0.12) | 3.04 (0.13) | 3.06 (0.13) |
| Transverse temporal cortex | 2.78 (0.21) | 2.76 (0.13) | 2.76 (0.17) | 2.76 (0.15) | 2.75 (0.16) | 2.77 (0.16) |
| Postcentral gyrus | 2.29 (0.13) | 2.31 (0.12) | 2.3 (0.16) | 2.32 (0.1) | 2.3 (0.13) | 2.33 (0.13) |
| Medial prefrontal cortex | 2.81 (0.09) | 2.82 (0.09) | 2.83 (0.11) | 2.8 (0.08) | 2.81 (0.09) | 2.8 (0.09) |
| Posterior cingulate cortex | 2.58 (0.1) | 2.6 (0.1) | 2.61 (0.12) | 2.6 (0.1) | 2.6 (0.1) | 2.61 (0.1) |
| Precuneus | 2.68 (0.11) | 2.67 (0.09) | 2.67 (0.1) | 2.68 (0.08) | 2.66 (0.1) | 2.68 (0.09) |
| Inferior parietal cortex | 2.72 (0.11) | 2.71 (0.11) | 2.71 (0.11) | 2.74 (0.09) | 2.73 (0.11) | 2.74 (0.1) |
| Parahippocampal gyrus | 2.85 (0.24) | 2.83 (0.24) | 2.88 (0.2) | 2.87 (0.23) | 2.78 (0.2) | 2.84 (0.2) |
| Inferior temporal cortex | 2.9 (0.1) | 2.98 (0.15) | 2.99 (0.11) | 3 (0.11) | 2.96 (0.12) | 2.98 (0.11) |
| Fusiform gyrus | 2.83 (0.12) | 2.89 (0.11) | 2.89 (0.09) | 2.88 (0.1) | 2.87 (0.1) | 2.89 (0.1) |
| Insula cortex | 3.11 (0.17) | 3.14 (0.14) | 3.17 (0.14) | 3.15 (0.11) | 3.15 (0.12) | 3.16 (0.13) |
| Dorsal anterior cingulate cortex | 2.76 (0.12) | 2.78 (0.12) | 2.8 (0.15) | 2.75 (0.12) | 2.76 (0.13) | 2.75 (0.12) |
| Supramarginal gyrus | 2.82 (0.13) | 2.81 (0.11) | 2.82 (0.14) | 2.83 (0.11) | 2.81 (0.12) | 2.82 (0.12) |
| Dorsal prefrontal cortex | 2.85 (0.11) | 2.83 (0.09) | 2.84 (0.1) | 2.84 (0.09) | 2.84 (0.1) | 2.85 (0.1) |
| Posterior parietal cortex | 2.73 (0.11) | 2.73 (0.09) | 2.73 (0.1) | 2.75 (0.08) | 2.73 (0.1) | 2.75 (0.09) |
| Intraparietal cortex | 2.6 (0.11) | 2.6 (0.1) | 2.61 (0.11) | 2.62 (0.09) | 2.61 (0.1) | 2.62 (0.1) |
| Ventral frontal cortex | 2.83 (0.1) | 2.83 (0.08) | 2.84 (0.11) | 2.83 (0.07) | 2.83 (0.09) | 2.83 (0.08) |
| Hippocampus | 7693.47 (833.57) | 8111.12 (868.04) | 7657.36 (740.41) | 8181.24 (773.96) | 7886.49 (770.68) | 8227.76 (771.31) |
| Amygdala | 3427.58 (463.17) | 3526.14 (471.52) | 3341 (316.08) | 3563.87 (464.98) | 3434.66 (415.69) | 3560.92 (416.49) |
| Thalamus | 14338.35 (1126.79) | 14854.59 (1600.92) | 14367.42 (1422.54) | 15007.97 (1393.97) | 14757.28 (1418.29) | 15240.44 (1392.66) |
| Cerebellum | 135917.1 (12730.86) | 143792.05 (16849.1) | 141292.12 (14362.17) | 146131.42 (14731.37) | 141298.14 (13888.87) | 147383.04 (13655) |
| Caudate | 107683.91 (10772.04) | 114116.95 (13608.18) | 112113.42 (11275.25) | 115754.37 (12369.74) | 111119.45 (11237.67) | 116301.94 (11213.69) |
| Putamen | 10286.78 (1054.59) | 10711.17 (1101.2) | 10236.46 (1258.15) | 10764.64 (1210.68) | 10444.95 (1121.83) | 10786.54 (1108.07) |

Data are presented as mean (SD). Low cognitive, low cognitive performance; Typical, typical performance. Low cognitive performance is defined as total cognition composite scores <–1 SD and >–2 SD. Typical performance is defined as total cognition composite scores: ≥-1 SD.

## Table A.3 Sensitivity analysis: Comparison of brain-hubs at 9–10 years of age between low cognitive performance and typical performance in very-preterm groups

| **Brain regions** | **Excluding poor intrauterine growth** | **Excluding neonatal complications** |
| --- | --- | --- |
|  | **Low cognitive (n=19) vs Typical (n=45)** | **Low cognitive (n=19) vs Typical (n=47)** |
| Pericalcarine cortex | -0.04 (-0.61 - 0.53) | -0.07 (-0.66 - 0.51) |
| lateral occipital cortex | -0.09 (-0.64 - 0.46) | -0.16 (-0.74 - 0.41) |
| Cuneus | -0.17 (-0.74 - 0.40) | -0.11 (-0.69 - 0.47) |
| Superior temporal cortex | -0.17 (-0.75 - 0.41) | -0.29 (-0.86 - 0.27) |
| Transverse temporal cortex | 0.17 (-0.43 - 0.76) | 0.01 (-0.59 - 0.61) |
| Postcentral gyrus | -0.18 (-0.77 - 0.40) | -0.33 (-0.91 - 0.25) |
| Medial prefrontal cortex | -0.26 (-0.83 - 0.31) | -0.25 (-0.83 - 0.33) |
| Posterior cingulate cortex | -0.09 (-0.68 - 0.49) | -0.12 (-0.70 - 0.46) |
| Precuneus | 0.24 (-0.36 - 0.83) | 0.20 (-0.40 - 0.80) |
| Inferior parietal cortex | 0.25 (-0.32 - 0.83) | 0.14 (-0.45 - 0.73) |
| Parahippocampal gyrus | 0.04 (-0.55 - 0.63) | 0.12 (-0.48 - 0.71) |
| Inferior temporal cortex | -0.45 (-1.01 - 0.10)^‡^ | -0.60 (-1.17 - -0.03)* |
| Fusiform gyrus | -0.54 (-1.12 - 0.04)^‡^ | -0.61 (-1.19 - -0.03)* |
| Insula cortex | -0.01 (-0.58 - 0.56) | -0.12 (-0.7 - 0.47) |
| Dorsal anterior cingulate cortex | -0.28 (-0.85 - 0.30) | -0.26 (-0.84 - 0.31) |
| Supramarginal gyrus | 0.31 (-0.26 - 0.88) | 0.22 (-0.37 - 0.8) |
| Dorsal prefrontal cortex | 0.17 (-0.42 - 0.76) | 0.06 (-0.53 - 0.65) |
| Posterior parietal cortex | 0.29 (-0.29 - 0.88) | 0.2 (-0.40 - 0.79) |
| Intraparietal cortex | 0.18 (-0.41 - 0.78) | 0.06 (-0.54 - 0.66) |
| Ventral frontal cortex | 0.06 (-0.52 - 0.64) | -0.07 (-0.66 - 0.51) |
| Hippocampus | 0.13 (-0.31 - 0.57) | -0.07 (-0.51 - 0.36) |
| Amygdala | 0.42 (-0.02 - 0.85) ^‡^ | 0.35 (-0.06 - 0.76)^‡^ |
| Thalamus | 0.36 (-0.01 - 0.73) | 0.22 (-0.13 - 0.58) |
| Cerebellum | 0.19 (-0.2 - 0.58) | 0.15 (-0.22 - 0.52) |
| Caudate | 0.15 (-0.25 - 0.55) | 0.13 (-0.25 - 0.52) |
| Putamen | 0.07 (-0.44 - 0.58) | -0.04 (-0.52 - 0.44) |

Data are presented as β (95%CI). Significant regions are shown using asterisk (p <0.05). ^‡^(p <0.10)

Abbreviations: Low cognitive, low cognitive performance; Typical, typical performance.

Low cognitive performance is defined as total cognition composite scores <–1 SD and >–2 SD. Typical performance is defined as total cognition composite scores: ≥-1 SD.

Table A.4 Sensitivity analysis: Comparison of brain-hubs at 9–10 years of age between low cognitive performance and typical performance in moderately-preterm groups

| **Brain regions** | **Excluding poor intrauterine growth** | **Excluding neonatal complications** |
| --- | --- | --- |
|  | **Low cognitive (n=33) vs Typical (n=98)** | **Low cognitive (n=33) vs Typical (n=102)** |
| Pericalcarine cortex | 0.11 (-0.29 - 0.51) | 0.24 (-0.17 - 0.64) |
| lateral occipital cortex | 0.03 (-0.35 - 0.42) | -0.04 (-0.42 - 0.34) |
| Cuneus | 0.03 (-0.39 - 0.45) | -0.08 (-0.49 - 0.32) |
| Superior temporal cortex | 0.18 (-0.23 - 0.59) | 0.10 (-0.31 - 0.52) |
| Transverse temporal cortex | 0.14 (-0.27 - 0.56) | 0.18 (-0.24 - 0.59) |
| Postcentral gyrus | -0.06 (-0.48 - 0.36) | -0.11 (-0.52 - 0.30) |
| Medial prefrontal cortex | 0.31 (-0.10 - 0.73) | 0.16 (-0.25 - 0.58) |
| Posterior cingulate cortex | 0.06 (-0.36 - 0.47) | 0.05 (-0.37 - 0.46) |
| Precuneus | -0.03 (-0.44 - 0.39) | -0.03 (-0.45 - 0.39) |
| Inferior parietal cortex | -0.09 (-0.51 - 0.33) | -0.27 (-0.69 - 0.15) |
| Parahippocampal gyrus | -0.03 (-0.45 - 0.39) | 0.04 (-0.38 - 0.45) |
| Inferior temporal cortex | 0.16 (-0.25 - 0.58) | 0.08 (-0.34 - 0.5) |
| Fusiform gyrus | 0.28 (-0.13 - 0.69) | 0.2 (-0.21 - 0.61) |
| Insula cortex | 0.18 (-0.23 - 0.59) | 0.19 (-0.23 - 0.6) |
| Dorsal anterior cingulate cortex | 0.42 (0.01 - 0.83)* | 0.27 (-0.14 - 0.69) |
| Supramarginal gyrus | 0.17 (-0.25 - 0.6) | 0.04 (-0.39 - 0.46) |
| Dorsal prefrontal cortex | 0.13 (-0.28 - 0.55) | -0.01 (-0.43 - 0.41) |
| Posterior parietal cortex | 0.001 (-0.42 - 0.42) | -0.14 (-0.56 - 0.28) |
| Intraparietal cortex | -0.01 (-0.42 - 0.41) | -0.19 (-0.61 - 0.23) |
| Ventral frontal cortex | 0.15 (-0.26 - 0.56) | 0.06 (-0.35 - 0.47) |
| Hippocampus | -0.49 (-0.76 - -0.22)* | -0.23 (-0.51 - 0.04) ^‡^ |
| Amygdala | -0.31 (-0.66 - 0.03) | -0.24 (-0.58 - 0.1) |
| Thalamus | -0.14 (-0.4 - 0.13) | -0.11 (-0.36 - 0.14) |
| Cerebellum | -0.02 (-0.32 - 0.27) | 0.06 (-0.23 - 0.36) |
| Caudate | 0.01 (-0.3 - 0.31) | 0.11 (-0.19 - 0.41) |
| Putamen | -0.21 (-0.52 - 0.11) | -0.28 (-0.6 - 0.04) |

Data are presented as β (95%CI). Significant regions are shown using asterisk (p <0.05). ^‡^(p <0.10)

Abbreviations: Low cognitive, low cognitive performance; Typical, typical performance. Low cognitive performance is defined as total cognition composite scores <–1 SD and >–2 SD. Typical performance is defined as total cognition composite scores: ≥-1 SD.

## Table A.5 Interaction effects of cognitive performance and socioeconomic status on brain hubs in preterm children aged 9-10 years

| **Brain regions** | **Very-preterm group** | | **Moderately-preterm group** | |
| --- | --- | --- | --- | --- |
|  | **β (95%CI)** | **p** | **β (95%CI)** | **p** |
| Pericalcarine cortex | -0.11 (-0.54 - 0.31) | 0.59 | 0.16 (-0.1 - 0.42) | 0.23 |
| lateral occipital cortex | 0.2 (-0.2 - 0.6) | 0.33 | -0.08 (-0.33 - 0.17) | 0.54 |
| Cuneus | -0.18 (-0.6 - 0.24) | 0.40 | 0.13 (-0.14 - 0.4) | 0.35 |
| Superior temporal cortex | 0.09 (-0.32 - 0.5) | 0.67 | 0 (-0.28 - 0.27) | 0.98 |
| Transverse temporal cortex | 0.02 (-0.42 - 0.46) | 0.94 | 0.28 (0.01 - 0.55) | 0.05 |
| Postcentral gyrus | 0.25 (-0.17 - 0.67) | 0.24 | 0.02 (-0.26 - 0.29) | 0.91 |
| Medial prefrontal cortex | -0.03 (-0.46 - 0.39) | 0.88 | 0.03 (-0.24 - 0.31) | 0.82 |
| Posterior cingulate cortex | -0.01 (-0.44 - 0.43) | 0.98 | 0.02 (-0.26 - 0.3) | 0.89 |
| Precuneus | 0.11 (-0.33 - 0.55) | 0.62 | 0.13 (-0.15 - 0.41) | 0.37 |
| Inferior parietal cortex | -0.01 (-0.44 - 0.41) | 0.95 | 0.04 (-0.24 - 0.31) | 0.80 |
| Parahippocampal gyrus | 0.31 (-0.13 - 0.74) | 0.16 | -0.01 (-0.29 - 0.27) | 0.95 |
| Inferior temporal cortex | -0.15 (-0.56 - 0.27) | 0.49 | -0.06 (-0.33 - 0.22) | 0.69 |
| Fusiform gyrus | 0.05 (-0.38 - 0.47) | 0.83 | 0.15 (-0.13 - 0.42) | 0.29 |
| Insula cortex | 0.3 (-0.13 - 0.72) | 0.17 | 0.08 (-0.18 - 0.35) | 0.53 |
| Dorsal anterior cingulate cortex | -0.01 (-0.44 - 0.42) | 0.95 | 0.03 (-0.25 - 0.31) | 0.83 |
| Supramarginal gyrus | 0.1 (-0.32 - 0.52) | 0.63 | 0.15 (-0.13 - 0.42) | 0.30 |
| Dorsal prefrontal cortex | 0.08 (-0.35 - 0.51) | 0.71 | 0.00 (-0.28 - 0.28) | 1.00 |
| Posterior parietal cortex | 0.06 (-0.38 - 0.49) | 0.80 | 0.09 (-0.19 - 0.37) | 0.53 |
| Intraparietal cortex | 0.05 (-0.39 - 0.48) | 0.83 | 0.05 (-0.22 - 0.33) | 0.69 |
| Ventral frontal cortex | 0.05 (-0.38 - 0.48) | 0.82 | 0.09 (-0.18 - 0.37) | 0.50 |
| Hippocampus | -0.16 (-0.46 - 0.15) | 0.30 | -0.04 (-0.22 - 0.15) | 0.70 |
| Amygdala | -0.17 (-0.46 - 0.12) | 0.24 | -0.2 (-0.42 - 0.02) | 0.07 |
| Thalamus | 0.02 (-0.23 - 0.28) | 0.86 | -0.21 (-0.37 - -0.05) | 0.01 |
| Cerebellum | -0.06 (-0.32 - 0.21) | 0.68 | -0.1 (-0.3 - 0.09) | 0.31 |
| Caudate | -0.02 (-0.3 - 0.26) | 0.89 | -0.1 (-0.29 - 0.1) | 0.34 |
| Putamen | -0.3 (-0.64 - 0.05) | 0.09 | -0.04 (-0.25 - 0.16) | 0.70 |

Here the β (95%CI) reflect the interaction effects of cognitive performance and socioeconomic status on brain hubs.

## Table A.6 Brain hubs comparison at 9-10 years of age between full-term children with low cognitive performance and typical performance

| **Brain hubs** | **Full-term children with low cognitive performance vs. typical performance** | |
| --- | --- | --- |
|  | **β (95% CI)** | **p** |
| Pericalcarine cortex | -0.15 (-0.22 - -0.09) | <0.001 |
| lateral occipital cortex | -0.17 (-0.22 - -0.11) | <0.001 |
| Cuneus | -0.15 (-0.21 - -0.08) | <0.001 |
| Superior temporal cortex | -0.06 (-0.13 – 0.001) | 0.06 |
| Transverse temporal cortex | -0.03 (-0.10 - 0.03) | 0.36 |
| Postcentral gyrus | -0.15 (-0.21 - -0.09) | <0.001 |
| Medial prefrontal cortex | 0.07 (0.001 - 0.13) | 0.04 |
| Posterior cingulate cortex | -0.02 (-0.09 - 0.05) | 0.59 |
| Precuneus | -0.06 (-0.13 – 0.001) | 0.07 |
| Inferior parietal cortex | -0.08 (-0.14 - -0.01) | 0.02 |
| Parahippocampal gyrus | -0.17 (-0.23 - -0.10) | <0.001 |
| Inferior temporal cortex | -0.12 (-0.19 - -0.06) | <0.001 |
| Fusiform gyrus | -0.1 (-0.17 - -0.04) | <0.001 |
| Insula cortex | -0.03 (-0.10 - 0.03) | 0.30 |
| Dorsal anterior cingulate cortex | 0.04 (-0.02 - 0.11) | 0.19 |
| Supramarginal gyrus | -0.08 (-0.14 - -0.02) | 0.01 |
| Dorsal prefrontal cortex | 0.03 (-0.04 - 0.09) | 0.40 |
| Posterior parietal cortex | -0.08 (-0.14 - -0.02) | 0.01 |
| Intraparietal cortex | -0.07 (-0.13 – -0.001) | 0.04 |
| Ventral frontal cortex | 0.04 (-0.03 - 0.10) | 0.25 |
| Hippocampus | -0.05 (-0.10 - -0.01) | 0.02 |
| Amygdala | 0.02 (-0.03 - 0.07) | 0.40 |
| Thalamus | 0.01 (-0.03 - 0.05) | 0.50 |
| Cerebellum | -0.07 (-0.12 - -0.02) | <0.001 |
| Caudate | -0.09 (-0.13 - -0.04) | <0.001 |
| Putamen | -0.02 (-0.08 - 0.03) | 0.40 |

# eFigures


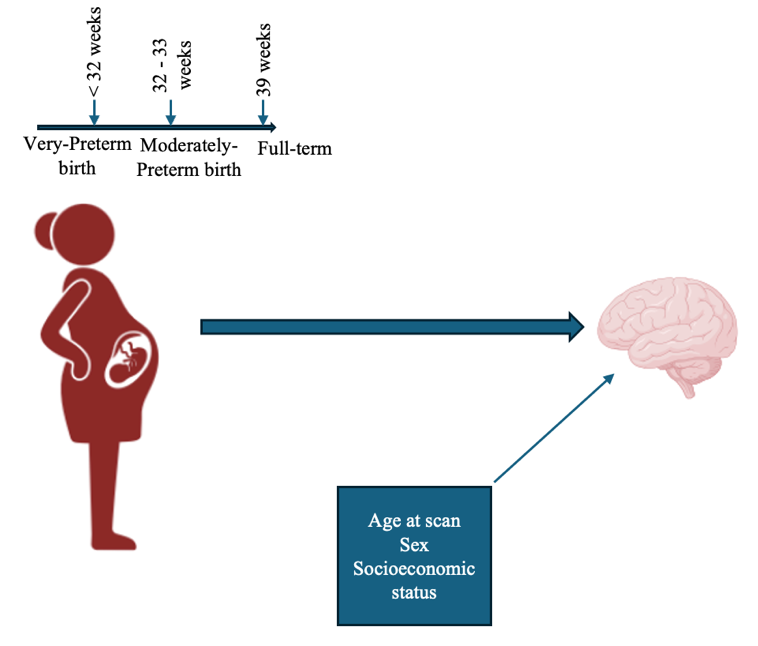


## Fig A.1 Covariates selected based on their association with brain outcomes

**
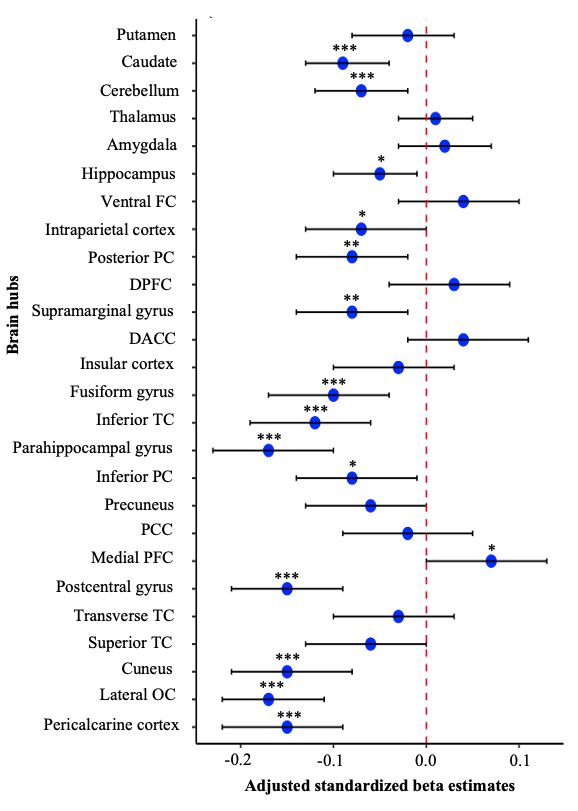
**

Fig A.2 Forest plot comparing brain hubs between full-term children with low cognitive performance and full-term with typical performance. Black lines represent confidence intervals (CIs), while blue dots indicate adjusted standardized beta estimates for each comparison. Low cognitive performance is defined as total cognition composite scores <–1 SD and >–2 SD. Typical performance is defined as total cognition composite scores: ≥-1 SD.

Abbreviations: FC, frontal cortex; PC, parietal cortex; DLFC, dorsolateral frontal cortex; DACC, dorsal anterior cingulate cortex; TC, temporal cortex; PCC, posterior cingulate cortex; PFC, prefrontal cortex; and OC, occipital cortex. *p < 0.05; **p < 0.01; ***p < 0.001.

**
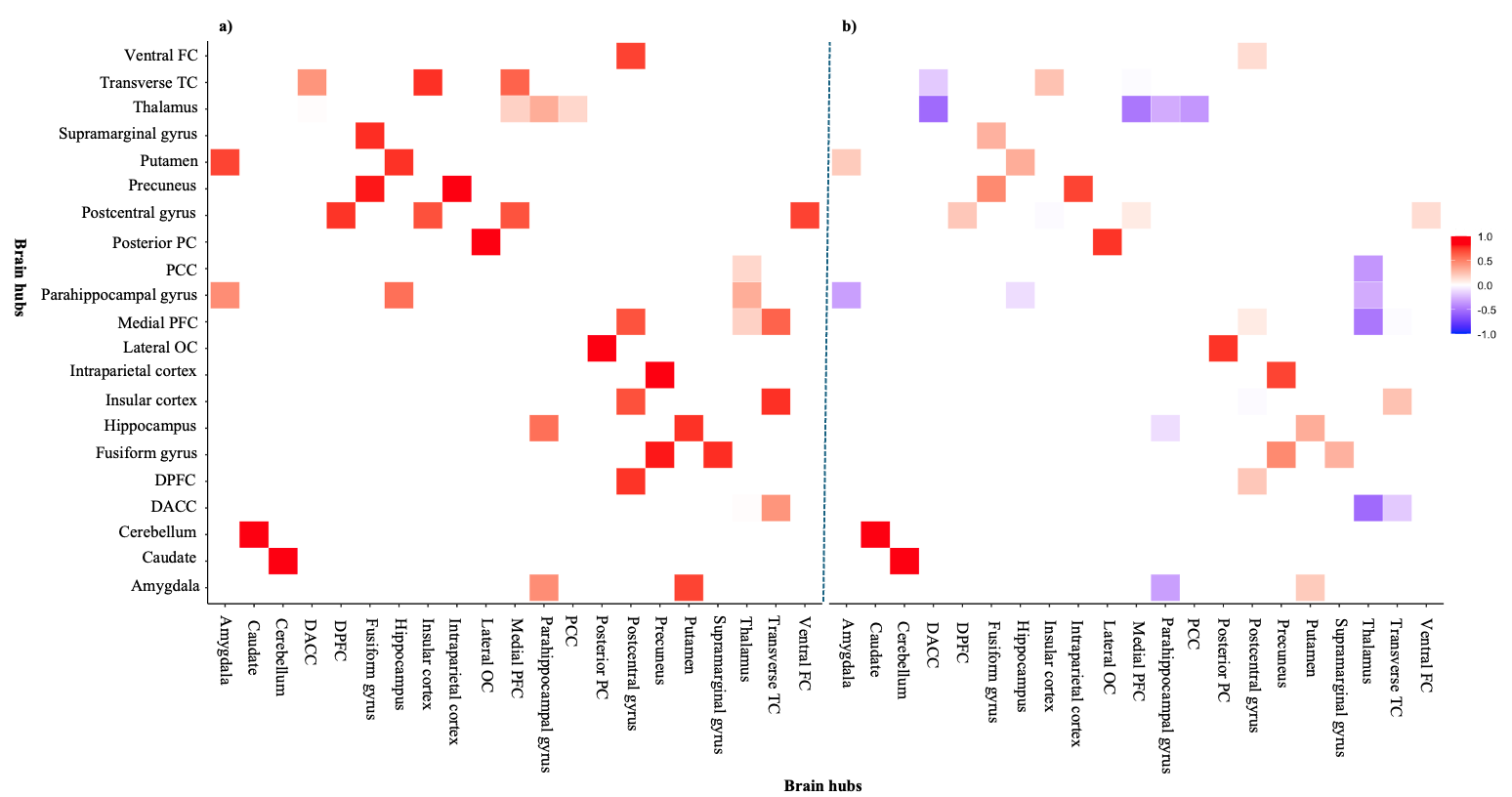
**

Fig A.3 Partial correlation of structural associations between brain hubs in very-preterm children grouped by cognitive performance at 9-10 years of age. **(a)** Low cognitive performance group exhibited stronger structural associations compared to **(b)** the typical performance group. Low cognitive performance is defined as total cognition composite scores <–1 SD and >–2 SD. Typical performance is defined as total cognition composite scores: ≥-1 SD. All associations with p-values below 0.05 are presented (uncorrected) and none of these associations were statistically significant after multiple comparison corrections. The colour gradient indicates the direction and magnitude of associations (red: positive, purple: negative).

**
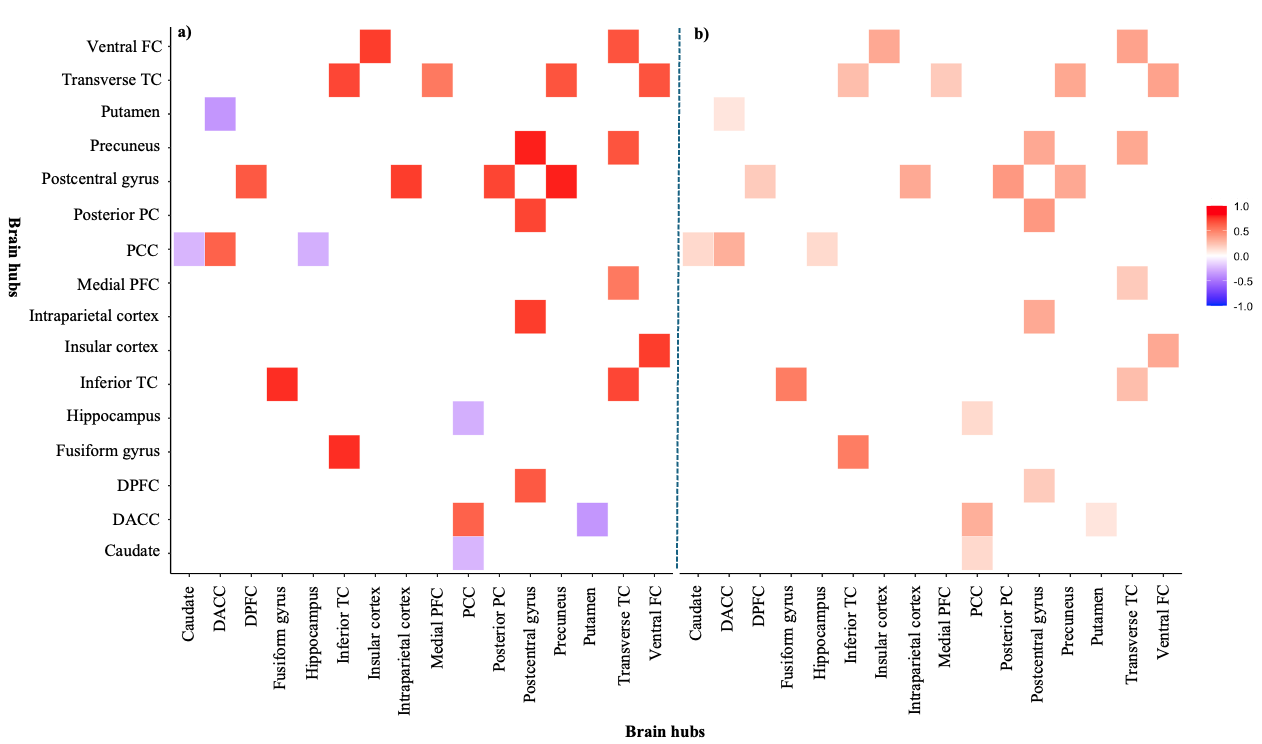
**

Fig A.4 Partial correlations of structural associations between brain hubs in moderately-preterm children stratified by cognitive performance at 9-10 years of age. (**a)** Low cognitive performance group exhibited stronger structural associations compared to **(b)** the typical performance group. Low cognitive performance is defined as total cognition composite scores <–1 SD and >–2 SD. Typical performance is defined as total cognition composite scores: ≥-1 SD. All associations with p-values below 0.05 are presented (uncorrected); however, none of these except for those between precuneus and postcentral gyrus survived multiple comparison corrections. The colour gradient indicates the strength and direction of partial correlations, with red representing positive associations and purple representing negative associations.

**
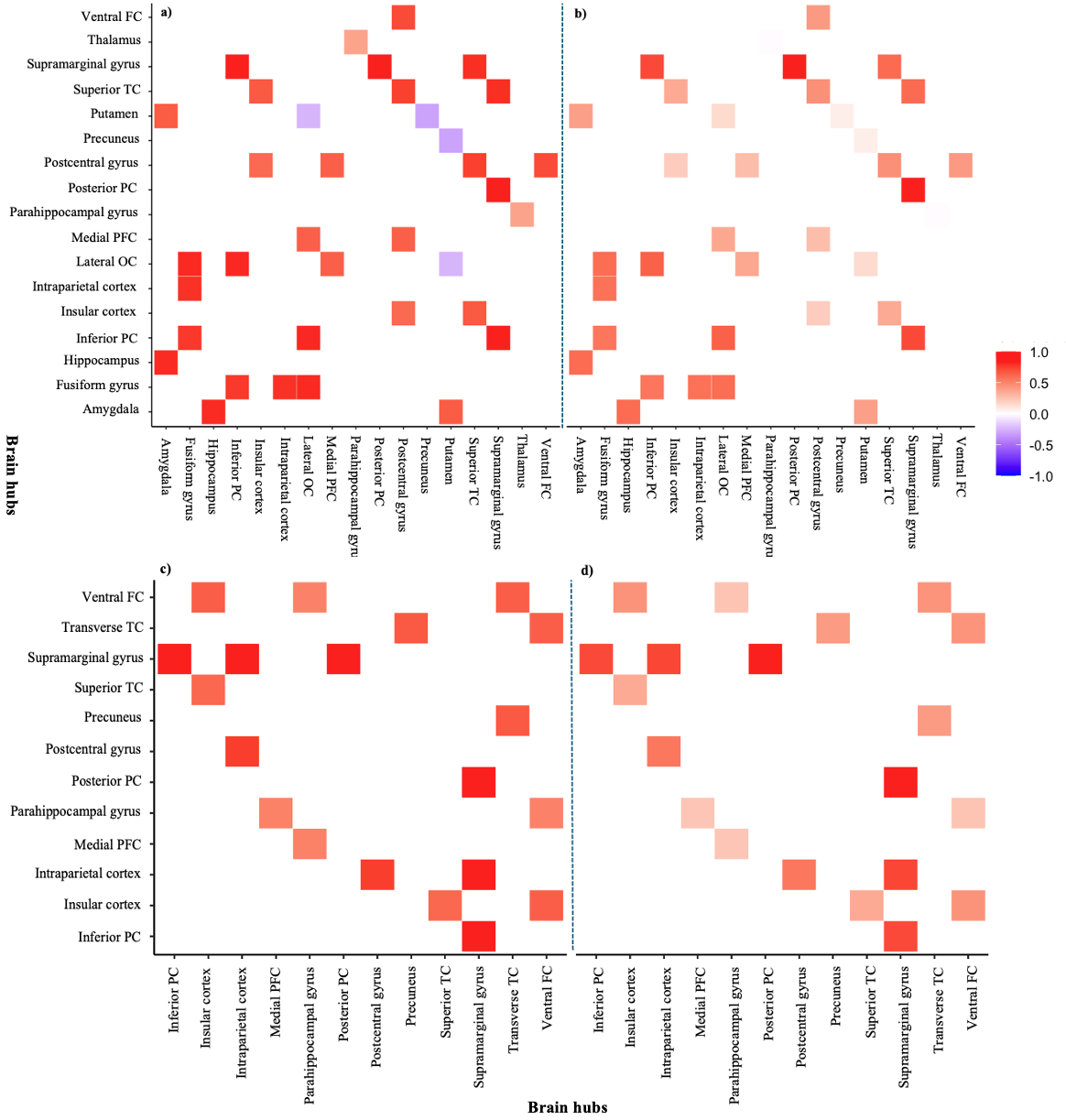
**

Fig A.5 Partial correlations of structural associations between brain hubs in 9-10-year-old children, stratified by cognitive performance and gestational age groups. **Top row:** Shows results for very-preterm children with low cognitive performance group **(a)** and full-term children with typical performance **(b)**. B**ottom** **row:** Displays results for moderately-preterm children with low cognitive performance group **(c)** and full-term children with typical performance **(d)**.

Compared to full-term children with typical performance, both very-preterm and moderately-preterm children with low cognitive performance group showed stronger structural associations between pairs of brain regions. Low cognitive performance is defined as total cognition composite scores <–1 SD and >–2 SD. Typical performance is defined as total cognition composite scores: ≥-1 SD.

All associations with p-values below 0.05 are presented (uncorrected) and none of these associations were statistically significant after multiple comparison corrections. The colour gradient indicates the strength and direction of partial correlations, with red representing positive associations and purple representing negative associations.

**
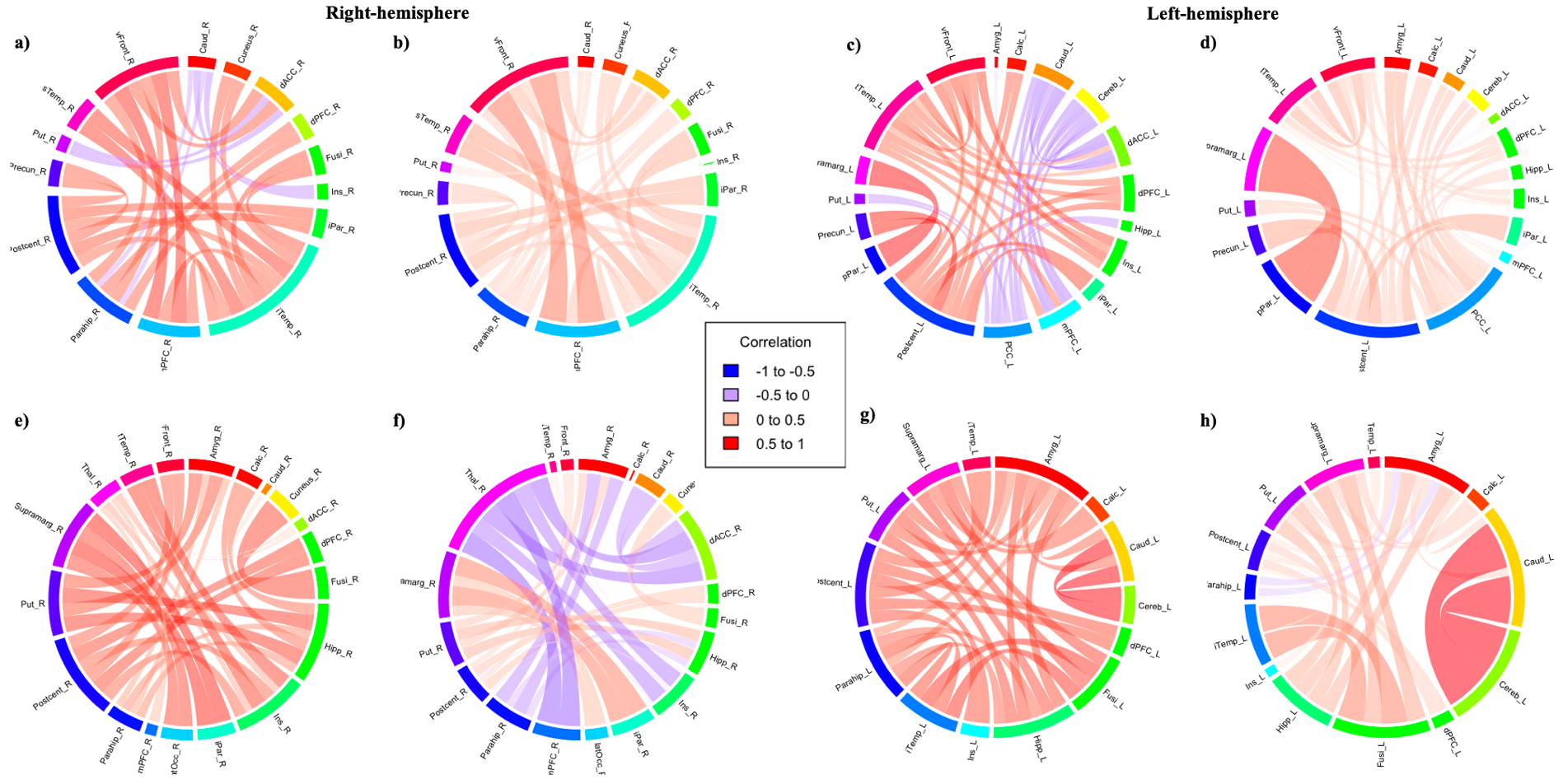
**

Fig A.6 Chord diagrams illustrating structural covariance patterns within hemispheres in 9-10-year-old children. **Top (a-d)**: Very preterm children with low cognitive performance had stronger within-hemisphere structural associations in the right **(a)** and left **(c)** hemispheres compared to those with typical performance **(b and d)**. **Bottom (e-h)**: Moderately preterm children with low cognitive performance had stronger within-hemisphere structural associations in the right **(e)** and left **(g)** hemispheres compared to their peers with typical performance **(f and h)**. All associations with p-values below 0.05 are presented (uncorrected). Low cognitive performance is defined as total cognition composite scores <–1 SD and >–2 SD. Typical performance is defined as total cognition composite scores: ≥-1 SD.

Abbreviations: Supramarg (Supramarginal Gyrus), Calc (Calcarine), Precun (Precuneus), latOcc (Lateral Occipital), Fusi (Fusiform), Amyg (Amygdala), iTemp (Inferior Temporal), Postcent (Postcentral Gyrus), tTemp (Transverse Temporal), Ins (Insula), dPFC (Dorsal Prefrontal Cortex), sTemp (Superior Temporal), mPFC (Medial Prefrontal Cortex), PCC (Posterior Cingulate Cortex), iPar (Inferior Parietal), Parahip (Parahippocampal Gyrus), Hipp (Hippocampus), Thal (Thalamus), dACC (Dorsal Anterior Cingulate Cortex), pPar (Posterior Parietal Cortex), vFront (Ventral Frontal Cortex), Put (Putamen), Caud (Caudate Nucleus), Cereb (Cerebellum).

**
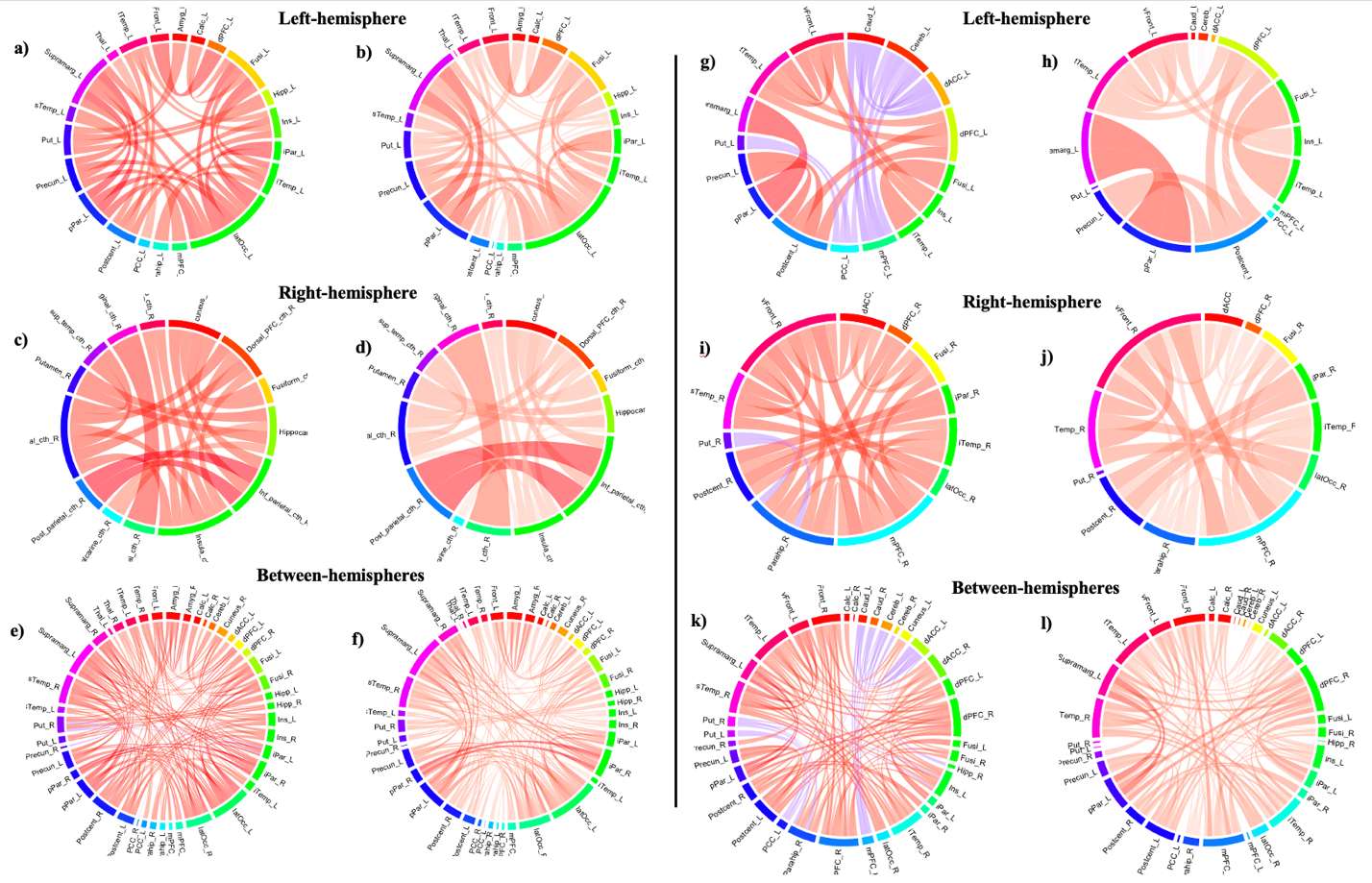
**

Fig A.7 Chord diagrams illustrating structural covariance patterns within and between hemispheres in 9-10-year-old children**.** **Left:** Very preterm children with low cognitive performance **(a, c, and e)** had stronger within-and between hemisphere structural associations compared to full-term children with typical performance **(b, d, and f)**. **Right:** Moderately preterm children with low cognitive performance **(g, i, and k)** had stronger within-and between hemisphere structural associations compared to full-term children with typical performance **(h, j, and l)**. All associations with p-values below 0.05 are presented (uncorrected). Low cognitive performance is defined as total cognition composite scores <–1 SD and >–2 SD. Typical performance is defined as total cognition composite scores: ≥-1 SD.

Abbreviations: Supramarg (Supramarginal Gyrus), Calc (Calcarine), Precun (Precuneus), latOcc (Lateral Occipital), Fusi (Fusiform), Amyg (Amygdala), iTemp (Inferior Temporal), Postcent (Postcentral Gyrus), tTemp (Transverse Temporal), Ins (Insula), dPFC (Dorsal Prefrontal Cortex), sTemp (Superior Temporal), mPFC (Medial Prefrontal Cortex), PCC (Posterior Cingulate Cortex), iPar (Inferior Parietal), Parahip (Parahippocampal Gyrus), Hipp (Hippocampus), Thal (Thalamus), dACC (Dorsal Anterior Cingulate Cortex), pPar (Posterior Parietal Cortex), vFront (Ventral Frontal Cortex), Put (Putamen), Caud (Caudate Nucleus), Cereb (Cerebellum).

# References

1. Casey, B.J., et al., The Adolescent Brain Cognitive Development (ABCD) study: Imaging acquisition across 21 sites. Developmental Cognitive Neuroscience, 2018. **32**: p. 43-54.

2. Hagler, D.J., Jr., et al., Image processing and analysis methods for the Adolescent Brain Cognitive Development Study. Neuroimage, 2019. **202**: p. 116091.

3. Li, Y., et al., Rates of Incidental Findings in Brain Magnetic Resonance Imaging in Children. JAMA Neurol, 2021. **78**(5): p. 578-587.

4. Cornejo, M.D., et al., Image processing and analysis methods for the Adolescent Brain Cognitive Development Study. NeuroImage (Orlando, Fla.), 2019. **202**: p. 116091-116091.

5. Desikan, R.S., et al., An automated labeling system for subdividing the human cerebral cortex on MRI scans into gyral based regions of interest. Neuroimage, 2006. **31**(3): p. 968-980.

6. Fischl, B., FreeSurfer. Neuroimage, 2012. **62**(2): p. 774-781.

7. Fischl, B., et al., Whole brain segmentation: automated labeling of neuroanatomical structures in the human brain. Neuron, 2002. **33**(3): p. 341-355.

8. Nivins, S., et al., Long-term impact of digital media on brain development in children. Scientific Reports, 2024. **14**(1): p. 13030.

9. Nivins, S., et al., Size at birth predicts later brain volumes. Scientific Reports, 2023. **13**(1): p. 12446.

10. Kaczkurkin, A.N., A. Raznahan, and T.D. Satterthwaite, Sex differences in the developing brain: insights from multimodal neuroimaging. Neuropsychopharmacology, 2019. **44**(1): p. 71-85.

11. Liu, Z., et al., Resolving heterogeneity in schizophrenia through a novel systems approach to brain structure: individualized structural covariance network analysis. Molecular psychiatry, 2021. **26**(12): p. 7719-7731.

12. Nivins, S., N. Padilla, and U. Ådén, Preterm birth, Cognitive deficits, and brain development. 2024.

13. Lee, J.Y., et al., Altered asymmetries of the structural networks comprising the fronto-limbic brain circuitry of preterm infants. Sci Rep, 2021. **11**(1): p. 1318.

14. Csárdi, G. and T. Nepusz. The igraph software package for complex network research. 2006.
