# Supplementary material for: Distinct neural mechanisms underlying cognitive difficulties in preterm children born at different stages of prematurity
Source: Neuroimage Clin. 2025 Sep 3;48:103876. doi: 10.1016/j.nicl.2025.103876 (PMC12454289; doi:10.1016/j.nicl.2025.103876)
Supplement: Supplementary Data 2 [file mmc2.docx]

**Supplementary Material 2**

**Statistical Analysis Plan for Pre-registration**

**Title: Preterm birth, Cognitive deficits, and brain development**

Prepared by Samson Nivins with feedback and inputs from Ulrika Arden and Nelly Padilla

Draft v1 prepared on

2024 – 09 – 26

Draft v2 prepared on

2024 – 09 -27

Draft v3 prepared on

2024 – 10 – 02

Draft v4 prepared on

2024 – 10 - 04

Draft final prepared on

2024 – 10 – 07

Registration DOI: <https://doi.org/10.17605/OSF.IO/TG8BK>

**Study Information**

**Overall research question**

Why are children born preterm, particularly those born before 34 weeks, at a higher risk for cognitive deficits during mid-childhood?

This research question is based on our previous findings, where we observed that children born before 34 weeks had lower composite cognitive scores compared to their full-term peers at 9-10 years of age. This association was independent of genetic, maternal, neonatal, and child factors.

**Hypotheses**

Children born before 34 weeks with cognitive deficits will show mixed patterns of cortical thickness (i.e., thinner in primary sensory hubs and thicker in higher-order cognitive hubs) and smaller subcortical volumes at 10 years of age compared to both children born before 34 weeks without cognitive deficits and those born at term without cognitive deficits.

We expect higher structural covariance (stronger connection/coordination) in primary sensory networks and lower structural covariance (weaker connection/coordination) in higher-order networks in children born before 34 weeks with cognitive deficits.

We will extract individual structural covariance values only for the networks that show significant differences in the primary analysis and explore their relationship with behavioral outcomes, such as ASD and ADHD symptoms at 12 years of age.

**Study design**

The Adolescent Brain Cognitive Development (ABCD) study is a prospective, multicenter, longitudinal study of children from the U.S., recruited between 2016 and 2018. Children underwent magnetic resonance imaging (MRI) scans at 9-10 years of age and completed comprehensive cognitive assessments, including the NIH Toolbox, Little Man Task, and the Rey Auditory Verbal Learning Test.

Parents completed the Developmental History Questionnaire when the children were 9-10 years old. At 12 years of age, parents filled out the Child Behaviour Checklist (CBCL), and the Short Social Responsiveness Scale (SSRS). Data for this study will be obtained from the NIMH Data Archive, Curated Annual Release 5.1. Informed consent/assent was obtained from all participants by the ABCD team, and the study protocols were approved by the central Institutional Review Board at the University of California, San Diego.

For present analysis, we will use brain MRI data from the 9–10-year assessments and neurodevelopmental assessments from the 12-year follow-up.

**Sampling plan**

**Existing data**

Registration prior to analysis of the data

**Explanation of existing data**

Samson Nivins has previously worked with the ABCD data; however, he has not analysed the specific measures or variables included in this pre-registration, except for cognitive outcomes, which he has explored in a different study design.

**Sample size**

This longitudinal cohort consists of 11,875 children, enrolled at baseline (9-10 years) and followed up annually at 21 research sites across the United States. Children were excluded if they were born extremely preterm (< 28 weeks), had a birth weight below 1200g, were not proficient in English, had neurological problems, a history of seizures, or a contraindication for undergoing brain magnetic resonance imaging (MRI) scans.

Since ABCD cohort includes twins and siblings, we will randomly select one child per family to eliminate this source of bias.

**Brain outcomes**

All pre-processing image quality checks were performed by trained raters who assessed images for poor quality (i.e., motion artifacts, blurring, or ringing).

The structural MRI data were processed by the ABCD DAIC team using FreeSurfer and underwent post-quality control procedures. The output from FreeSurfer was then visually inspected by trained DAIC technicians, who rated images on a scale of zero to three in five categories: motion, intensity homogeneity, white matter underestimation, pial overestimation, and magnetic susceptibility artifacts.

From these ratings, an overall ‘1’ or ‘0’ score was generated. Children whose images failed quality control will be excluded from the present analyses.

**Variables**

All maternal and neonatal parameters are available (appropriate permission were obtained).

**Cognitive outcomes**

The neurocognitive battery, designed for completion in 70 minutes, began with Snellen vision chart to assess visual acuity, excluding children with legal blindness. The battery includes ten measures, seven from NIH Toolbox: Picture Vocabulary, Oral Reading Recognition, Pattern Comparison, Processing Speed, List-Sorting Working Memory, Picture Sequence Memory, Flanker, and Dimensional Change Card Sort. The ABCD study team also assessed Little Man Task (LMT) and Rey Auditory Verbal Learning Test (RAVLT). All tests were administered on an iPad with one-on-one monitoring by a research-assistant.

Cognitive deficit will be defined as a score between 1-2 standard deviation below the control group mean, based on the fully age-corrected composite score from the NIH Toolbox.

**Social Responsiveness Scale (SRS)**

Autistic traits will be assessed using SRS, which is primarily used to assess the severity of social difficulties in autistic and non-autistic children, and have been evaluated in a UK population-based sample of 5-to-8-year-old children. In ABCD sample, parents answered questionnaire. Total raw scores from children will be calculated.

**Child Behavior Checklist (CBCL)**

The parent/caregiver of the child completed the Child Behaviour Checklist (CBCL). The Child Behaviour Checklist has been used to determine the extent of behavioural anomalies amongst children and adolescents. It contains 113 items, which were rated on a three- point Likert scale, of which items corresponding to ADHD symptoms will be considered. According to the normative data of the CBCL, a t-score ≤ 59 indicates non-clinical symptoms, a t-score between 60 and 64 indicates that the child is at risk for problem behaviours, and a t-score ≥ 65 indicates clinical symptoms

**Outcomes**

Primary outcomes: Primary networks (sensory, visual, and limbic)

Secondary outcomes: Higher-order networks (salience network, dorsal attention network, default mode network, fronto-parietal network)

Brain hubs corresponding to the mentioned networks will be studied

Cortical thickness from DK atlas and subcortical volumes will be used.

**Exposure**

Moderately preterm (32 - 34 weeks)

Very preterm (< 32 weeks)

Full-term (39 – 40 weeks)

**Covariates**

Age at time of MR scan, Sex, Socio-economic status (maternal education, household income, and neighbor area index), and scanner sites. These variables were selected as they known to be associated with cognitive outcomes or brain development.

**Statistics**

Maternal and child characteristics will be summarized using means and standard deviations for continuous variables and frequencies with counts for categorical distributions.

To compare demographic and clinical characteristics between children born very preterm, born moderately preterm and born at term, independent samples t-test and χ^2^/Fisher exact tests will be employed.

**Analysis**

Linear regression model will be used to investigate whether brain structures (cortical thickness and subcortical volumes) differ between children with and without cognitive deficits (presented as standardized beta and 95% CI)

**Primary analysis:**

- Very preterm with cognitive deficits Vs. Very preterm without cognitive deficits
- Moderately preterm with cognitive deficits Vs. Moderately preterm without cognitive deficits

**Secondary analysis:**

- Very preterm with cognitive deficits Vs. full-term without cognitive deficits
- Moderately preterm with cognitive deficits Vs. full-term without cognitive deficits

All models will be adjusted for above-mentioned confounders/covariates.

**Sensitivity analysis:**

No neonatal complications (yes/no)

Poor intrauterine growth (birthweight-z score as proxy, < 1SD)

Environmental interaction (groups x SES)

**Constructing of Structural Covariance Networks (SCN):**

To investigate SCN, Pearson partial correlation analysis will be conducted on the brain hubs. This analysis will be performed separately for groups: children born preterm with cognitive deficits, children born preterm without cognitive deficits, and children born at-term with normal cognitive function.

In the first step, we will calculate correlation coefficients between each pair of regions within the specified volumes for each group. These correlations will be adjusted for potential confounding variables, including age, sex, socioeconomic status (SES). To control for multiple comparisons, FDR correction will be applied when assessing significant structural covariance between each pair of cortical regions.

In the second step, Fisher’s r-to-z transformation will be applied to the significant structural covariance connections to standardize the correlation coefficients.

Finally, we will assess between-group differences in cognitive structural covariance networks using a z-test. Specifically, we will compare the networks of preterm children with cognitive deficits against those of preterm children without cognitive deficits, and against those of term-born children with normal cognitive function.

**Association between SCNs and neurobehavioral scores**

To explore the relationship between altered networks and behavioural outcomes and alterations where significant group differences are identified, as described above, we will use Inter-Disciplinary Structural Covariance (IDSC) values. IDSC values represent each child's contribution to their overall group's structural covariance and serve as a measure of inter-regional association strength for that individual.
